# Benefit–Hematotoxicity Stratification in Patients with Triple-Negative Breast Cancer Receiving Platinum-Based Neoadjuvant Therapy: A Multitask Deep Learning Study

**DOI:** 10.3390/cancers18172842

**Published:** 2026-09-02

**Authors:** Hao Sun, Xinglu Zhou, Jian Liang, Yujie Shi, Bao Deng, Ziqi Guo, Tong Su, Binbin Guo, Lei Zhong

**Affiliations:** 1 Department of Breast Surgery, The Sixth Affiliated Hospital of Harbin Medical University, No. 998 Aiying Street, Songbei District, Harbin 150023, China; haosun@hrbmu.edu.cn (H.S.);; 2 Department of PET/CT Center, Harbin Medical University Cancer Hospital, No. 150 Haping Road, Nangang District, Harbin 150081, China; zhouxinglu@hrbmu.edu.cn; 3 The Second Affiliated Hospital of Harbin Medical University, No. 246 Xuefu Road, Nangang District, Harbin 150001, China; 4 Department of Ultrasound, The Sixth Affiliated Hospital of Harbin Medical University, No. 998 Aiying Street, Songbei District, Harbin 150023, China

**Keywords:** triple-negative breast cancer, platinum-based neoadjuvant therapy, pathological complete response, severe hematotoxicity, multitask learning

## Abstract

**Objectives:** Platinum-based neoadjuvant therapy can improve pathological response in triple-negative breast cancer (TNBC), but severe hematotoxicity may compromise treatment delivery. This study developed and validated a multitask learning framework to jointly predict pathological complete response (pCR) and severe hematotoxicity and to support benefit–hematotoxicity stratification. **Methods:** This multicenter retrospective study included 2060 consecutive patients with TNBC receiving platinum-based neoadjuvant therapy at three institutions. Patients were assigned to a training cohort (*n* = 1406), an internal validation cohort (*n* = 351), or an external validation cohort (*n* = 303). A multitask TabNet (MT-TabNet) model was developed using pretreatment clinical, pathological, imaging, laboratory, electrocardiographic, and planned treatment exposure variables to jointly estimate pCR and severe hematotoxicity. Model performance was evaluated using discrimination, calibration, decision curve analysis, and interpretability analyses. The predicted probabilities were further integrated into a utility-based framework for benefit–hematotoxicity stratification. **Results:** Overall, 639 patients (31.0%) achieved pCR, and 651 (31.6%) developed severe hematotoxicity. MT-TabNet achieved AUCs of 0.874, 0.842, and 0.819 for pCR prediction and 0.859, 0.846, and 0.818 for severe hematotoxicity prediction in the training, internal validation, and external validation cohorts, respectively. The model showed generally acceptable calibration and clinical net benefit. pCR prediction was predominantly associated with tumor-related characteristics, whereas severe hematotoxicity prediction was more strongly associated with planned treatment exposure and host-related laboratory indicators. Utility-based stratification showed progressively higher pCR rates and lower severe hematotoxicity rates from the low- to high-benefit groups across all three cohorts. Survival differed significantly among benefit groups for both progression-free survival (PFS) and overall survival (OS) in the training and internal validation cohorts; in the external validation cohort, OS differed significantly, whereas PFS showed a similar but nonsignificant trend. **Conclusions:** MT-TabNet enabled joint estimation of pCR and severe hematotoxicity in patients with TNBC receiving platinum-based neoadjuvant therapy. The utility-based framework integrated efficacy and toxicity predictions into clinically interpretable benefit–hematotoxicity stratification and warrants further prospective evaluation for individualized risk assessment and toxicity monitoring.

## Simple Summary

Platinum-based treatment is commonly given before surgery to patients with triple-negative breast cancer, but not all patients benefit from it, and some develop serious blood-related side effects. This study developed a multitask artificial intelligence model to estimate both the likelihood of pathological complete response after treatment and the risk of severe blood toxicity before treatment begins. By combining these two predictions, the model divided patients into groups with different expected benefit and toxicity profiles. This approach may help clinicians identify patients who are more likely to benefit from platinum-based treatment and those who may need closer monitoring or supportive care. The findings provide a practical framework for balancing treatment benefit and safety in patients with triple-negative breast cancer.

## 1. Introduction

Triple-negative breast cancer (TNBC) is characterized by aggressive biological behavior, a high risk of recurrence, and limited therapeutic targets [1,2]. For patients with high-risk early-stage or locally advanced TNBC, neoadjuvant therapy is an important component of multidisciplinary treatment [3]. It can reduce tumor burden, increase pathological response, facilitate breast-conserving surgery and axillary downstaging, and inform postoperative treatment decisions [4,5]. With the continued intensification of treatment strategies, neoadjuvant therapy for TNBC now commonly involves anthracycline- and taxane-based backbones combined with platinum agents or immunotherapy [6,7]. Among these options, platinum agents represent an important intensification strategy because of their established antitumor activity, clinical accessibility, and compatibility with standard chemotherapy backbones [8]. Previous studies have shown that adding platinum agents to standard neoadjuvant chemotherapy can further improve pathological response in selected patients; however, the magnitude of benefit varies substantially, and the optimal candidates and treatment strategies remain to be clarified [9,10,11].

Hematologic toxicity is a major safety concern that limits the delivery of platinum-based therapy [12]. Severe neutropenia, thrombocytopenia, anemia, and febrile neutropenia may increase the risks of infection, bleeding, transfusion, hospitalization, and anti-infective treatment [13,14]. These toxicities may also lead to treatment delays, dose reductions, or early discontinuation of platinum agents, thereby compromising treatment continuity, dose intensity, and the timing of subsequent surgery [15,16]. Therefore, treatment-limiting hematotoxicity is not merely a safety endpoint but also an important determinant of the clinical net benefit of platinum-based neoadjuvant therapy [17]. The suitability of platinum-based therapy depends on the balance between pathological benefit and hematotoxicity risk [18]. Evaluating pathological response or toxicity risk in isolation is insufficient to determine whether an individual patient is suitable for platinum intensification. Thus, a stratification framework that integrates both therapeutic benefit and hematologic risk is needed to support individualized treatment decision-making [19].

Although previous studies have used clinicopathological features, radiomics, pathology images, or machine learning methods to predict neoadjuvant treatment response in TNBC [20,21], and others have focused on chemotherapy-related adverse events [22], most existing models are centered on a single outcome. Such models cannot fully reflect the clinical reality that efficacy benefit and toxicity cost coexist in platinum-based neoadjuvant therapy. In the pretreatment decision-making setting, response-only prediction cannot inform treatment tolerance, whereas toxicity-only prediction cannot determine whether toxicity risk may be justified by potential pathological benefit. Therefore, models that integrate pathological benefit and treatment-limiting hematotoxicity within a unified framework are needed to generate clinically interpretable and decision-relevant patient stratification.

To address this need, we applied a TabNet-based multitask learning framework to jointly predict pathological response and severe hematotoxicity using structured clinical data. TabNet can capture nonlinear relationships among multidimensional clinical variables while retaining a degree of model interpretability through its attention-based feature selection mechanism [23]. The multitask framework enables simultaneous estimation of treatment efficacy and toxicity while allowing each outcome to retain its own predictive characteristics. In this study, we developed and evaluated this framework in multicenter cohorts of patients with TNBC receiving platinum-based neoadjuvant therapy. The model was designed to estimate individualized probabilities of pCR and severe hematotoxicity, identify predictors associated with each outcome, and provide an integrated basis for subsequent benefit–toxicity stratification.

## 2. Materials and Methods

### 2.1. Patients

**Figure 1:**
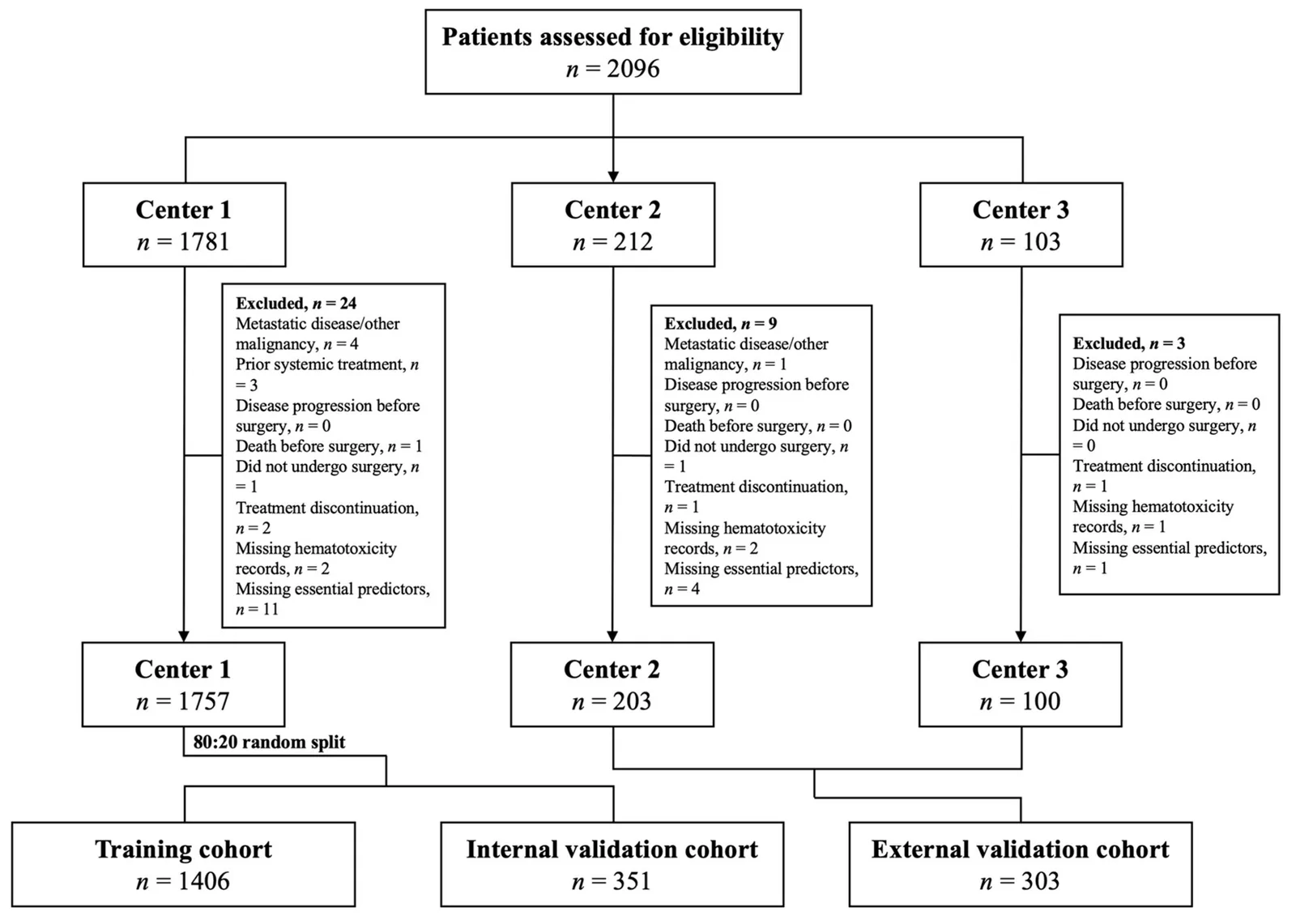
Flowchart of patient selection and cohort construction.

This multicenter retrospective cohort study screened 2096 consecutive patients with TNBC who received platinum-based neoadjuvant therapy at three institutions between March 2021 and December 2025. After excluding 36 patients according to the predefined eligibility criteria, 2060 patients were included in the final analysis. Harbin Medical University Cancer Hospital, the Second Affiliated Hospital of Harbin Medical University, and the Sixth Affiliated Hospital of Harbin Medical University were designated as Centers 1, 2, and 3, respectively. Patients from Center 1 constituted the model development cohort (*n* = 1757) and were randomly assigned to a training cohort (*n* = 1406) and an internal validation cohort (*n* = 351) using a prespecified 80:20 ratio using a computer-generated random sequence with a fixed random seed of 42. Patients from Centers 2 and 3 (*n* = 203 and *n* = 100, respectively) were collected during the same study period using identical inclusion and exclusion criteria and constituted the external validation cohort (*n* = 303). All cases were collected consecutively to reduce selection bias. The patient selection and cohort allocation process is shown in Figure 1.

All included patients received platinum-based neoadjuvant therapy, mainly consisting of a taxane plus carboplatin. A subset of patients additionally received sequential anthracycline/cyclophosphamide therapy and immunotherapy according to the treatment plan established before neoadjuvant therapy and clinical indications. These treatment exposures were planned before the initiation of neoadjuvant therapy. Accordingly, planned treatment exposure was treated as a pretreatment variable and included in the model. Treatment was generally administered in 3-week cycles for 4–8 cycles before surgery, with response assessments performed every 2–4 cycles. Specific treatment arrangements were determined by treating physicians according to contemporaneous guideline recommendations, patient condition, and treatment tolerance.

The inclusion criteria were as follows: (1) pathologically confirmed TNBC, defined as estrogen receptor (ER) and progesterone receptor (PR) expression in <1% of tumor cell nuclei by immunohistochemistry, and HER2-negative status defined as an immunohistochemistry (IHC) score of 0 or 1+, or an IHC score of 2+ with negative in situ hybridization (ISH); (2) receipt of platinum-based neoadjuvant therapy; (3) subsequent surgery with available pathological response assessment; and (4) availability of baseline clinical, imaging, laboratory, electrocardiographic, and treatment-related data required for model development. The exclusion criteria were as follows: (1) distant metastatic disease at diagnosis; (2) history of other malignancies; (3) prior systemic treatment for breast cancer before neoadjuvant therapy; (4) failure to undergo surgery or absence of pathological response assessment; (5) missing hematotoxicity records; and (6) missing essential baseline predictors used for model development. A summary of missing data and the corresponding reasons for missingness is provided in Supplementary Table S1.

The study protocol was approved by the Ethics Committee of the Sixth Affiliated Hospital of Harbin Medical University (Approval No. LC2024-052) and was conducted in accordance with the Declaration of Helsinki.

### 2.2. Data Collection and Preprocessing

Baseline data were collected before the initiation of platinum-based neoadjuvant therapy, and all variables used for prediction were available at this pretreatment time point. The collected variables included demographic and comorbidity information, tumor-related clinical and imaging characteristics, pathological markers, laboratory parameters, electrocardiographic measurements, and planned treatment exposure. Demographic and comorbidity variables included age, body mass index, hypertension, and diabetes. Tumor-related variables included clinical stage, tumor size, axillary lymph node status, tumor margin, and vascularity. Axillary lymph node status was determined from pretreatment axillary imaging assessments. Tumor margin and vascularity were obtained from pretreatment breast ultrasonography, with tumor margin categorized as well-defined or ill-defined and vascularity as low or high according to the original imaging reports. Pathological markers included Ki-67 expression and P53 status obtained from pathological assessment before neoadjuvant therapy. P53 status was recorded as positive or negative according to the original pretreatment pathological report. Ki-67 was recorded as a percentage and entered into the prediction models as a continuous variable; categorical groupings were used only for descriptive presentation. Laboratory parameters included liver and renal function, protein and glucose metabolism, hematological indices, inflammatory markers, and coagulation-related markers. QTc interval and heart rate were obtained from pretreatment electrocardiographic examinations. QTc status was categorized according to the original electrocardiographic assessment. Planned treatment exposure included planned anthracycline-containing therapy and immunotherapy. Because these assessments were completed before neoadjuvant therapy, subsequent pathological response and hematotoxicity outcomes were not yet known at the time of data collection or the original clinical, imaging, pathological, and electrocardiographic assessments (Supplementary Table S2).

Patients who did not undergo surgery or lacked pathological response assessment, those with missing hematotoxicity records, and those with missing essential baseline predictors were excluded during cohort assembly. Among the 2096 screened patients, 16 (0.8%) were excluded because of missing essential baseline predictors. Given the low proportion of missing baseline predictor data, complete-case analysis was performed without imputation. Continuous variables were standardized before model development, whereas categorical variables were converted into indicator variables. All preprocessing procedures were established using the training cohort and then applied to the internal and external validation cohorts, so that information from the validation cohorts was not used during model development.

### 2.3. Outcome Definitions

The two primary outcomes of the multitask learning framework were pathological benefit and severe hematotoxicity. Pathological benefit was defined as pathological complete response (pCR), corresponding to the absence of residual invasive cancer in both the breast and axillary lymph nodes after neoadjuvant therapy. Patients with residual invasive disease in either the breast or axillary lymph nodes were classified as non-pCR.

Severe hematotoxicity was defined as the occurrence of any CTCAE v5.0 grade 3–4 hematologic adverse event or febrile neutropenia during platinum-based neoadjuvant therapy. Hematologic adverse events included neutropenia, leukopenia, anemia, and thrombocytopenia. For patients with multiple hematologic events, the endpoint was coded once at the patient level. Treatment delay, dose reduction, early discontinuation, hospitalization, transfusion, or granulocyte colony-stimulating factor support related to hematotoxicity were additionally recorded to characterize treatment-limiting consequences.

Progression-free survival (PFS) and overall survival (OS) were evaluated as secondary prognostic outcomes. PFS was defined as the time from initiation of neoadjuvant therapy to disease progression, recurrence, or death from any cause. OS was defined as the time from initiation of neoadjuvant therapy to death from any cause. Outcome data were obtained from clinical records, follow-up visits, and routine telephone follow-up. Patients without the corresponding event were censored at the date of last follow-up.

### 2.4. Model Development, Evaluation, and Interpretability

A TabNet-based multitask model was developed to simultaneously estimate the probabilities of pCR and severe hematotoxicity using the pretreatment predictors described above. The model learned information common to both outcomes while generating separate probability estimates for pCR and severe hematotoxicity. Binary cross-entropy loss was used for each outcome, and the overall loss was calculated as the equally weighted sum of the two outcome-specific losses.

Model development and parameter selection were performed exclusively within the training cohort. Stratified five-fold cross-validation was used for hyperparameter selection, with stratification based on the joint distribution of pCR and severe hematotoxicity to maintain the distribution of both outcomes across folds. After hyperparameter selection, repeated stratified five-fold cross-validation with five repetitions was performed within the training cohort to generate out-of-fold predictions and assess model stability. All training-cohort performance metrics reported in this study were calculated from these repeated cross-validation out-of-fold predictions rather than from apparent predictions obtained after refitting the final model to the full training cohort. The original outcome distributions were retained without over-sampling, under-sampling, synthetic sample generation, or class weighting. Data standardization and categorical-variable coding were performed separately within each training fold and then applied to the corresponding validation fold.

Model parameters were optimized within a prespecified search space using five-fold cross-validation. For MT-TabNet, model selection was based on the mean validation binary cross-entropy across the two outcomes. For ST-TabNet and the comparator models, selection was based on the mean validation log loss for the corresponding outcome. Early stopping was used during TabNet training. The complete parameter search spaces and the final selected values are provided in Supplementary Table S3.

MT-TabNet was compared with ST-TabNet and three conventional prediction models: ridge-penalized logistic regression, random forest, and XGBoost. All models used the same prespecified pretreatment predictor set. After model selection, the final models were refitted using the full training cohort. The internal and external validation cohorts were then used only for model evaluation, with no further model fitting or parameter adjustment.

Predictive performance was evaluated in terms of discrimination, calibration, and clinical utility. Discrimination was assessed using the area under the receiver operating characteristic curve (AUC), sensitivity, specificity, positive predictive value, and negative predictive value. For threshold-dependent measures, classification thresholds were determined separately for each outcome from out-of-fold predictions in the training cohort by maximizing the Youden index and were subsequently fixed for application to the internal and external validation cohorts. AUCs were reported with 95% confidence intervals and were compared between MT-TabNet and the comparator models using the DeLong test. Calibration was evaluated using the Brier score, calibration intercept, calibration slope, and calibration curves based on deciles of predicted probability. Ninety-five percent confidence intervals for performance measures were estimated using 1000 bootstrap resamples. Clinical utility was evaluated using decision curve analysis.

To assess the effect of joint modeling, the performance of MT-TabNet was compared with that of the corresponding ST-TabNet model for each outcome. Differences in AUCs and Brier scores were used to evaluate changes in discrimination and overall prediction accuracy, while variation across repeated cross-validation runs was used to assess model stability.

Model interpretability was assessed using feature importance and SHapley Additive exPlanations (SHAP). Predictor contributions were examined separately for pCR and severe hematotoxicity to identify both shared and outcome-specific predictive patterns. Permutation-based feature ablation analysis was additionally performed by randomly permuting each predictor 100 times and measuring the resulting reduction in AUC. The mean AUC reduction was reported, with the 2.5th and 97.5th percentiles of the permutation distribution used as the 95% confidence interval.

### 2.5. Utility-Based Benefit–Hematotoxicity Stratification and Prognostic Validation

To integrate the two multitask predictions into an overall measure of treatment benefit and toxicity, a utility-based framework was constructed using the predicted probabilities generated by MT-TabNet. The utility score was defined as U = P(pCR) − λP(hematotoxicity), where a higher predicted probability of pCR increased the utility score and a higher predicted probability of severe hematotoxicity decreased it. The parameter λ represented the relative weight assigned to severe hematotoxicity when pathological benefit and toxicity were considered jointly. Accordingly, larger λ values placed greater weight on avoiding severe hematotoxicity, whereas smaller λ values placed relatively greater weight on achieving pathological response.

The data-derived reference λ was determined exclusively in the training cohort using training-cohort out-of-fold predictions. Achievement of pCR without severe hematotoxicity was defined as the favorable reference state, representing concordant benefit in both pathological response and treatment safety, whereas non-pCR with severe hematotoxicity was defined as the unfavorable reference state, representing concordant unfavorable status in both dimensions. These two joint outcomes represent clinically distinct states at opposite ends of the efficacy–toxicity spectrum and were therefore used as reference states for estimating λ.

For each candidate λ, separation between the favorable and unfavorable reference states was quantified using a standardized separation criterion that accounted for both between-state differences and within-state dispersion. Specifically, the criterion was calculated as the squared difference in mean utility scores between the two reference states divided by the sum of their corresponding within-state utility variances. Larger criterion values therefore indicated clearer separation between favorable and unfavorable efficacy–toxicity profiles.

Candidate λ values were evaluated over a prespecified range of 0 to 2.0. To improve the stability of λ estimation, the procedure was implemented using 500 bootstrap resamples of the training cohort. For each bootstrap sample, the standardized separation criterion was calculated across the candidate λ values. The mean criterion value across bootstrap samples was used to construct the optimization curve, and the bootstrap distribution at each λ was used to characterize uncertainty around the objective. The λ value corresponding to the maximum mean bootstrap separation criterion was selected as the reference λ and was subsequently fixed before application to the internal and external validation cohorts.

Based on the utility-score distribution of the entire training cohort, patients were stratified into low-, intermediate-, and high-benefit groups using the 25th and 75th percentiles as prespecified cutoffs. The reference λ and the corresponding numerical cutoffs were then fixed and applied unchanged to the internal and external validation cohorts. To evaluate the robustness of the stratification under different relative weightings of pathological benefit and severe hematotoxicity, sensitivity analyses were additionally performed using λ values of 0.5, 1.0, 1.5, and 2.0. For each weighting scenario, the corresponding stratification cutoffs were derived exclusively from the training cohort and subsequently fixed for application to the internal and external validation cohorts.

A favorable composite outcome was defined as achievement of pCR without severe hematotoxicity. The composite outcome was coded as 1 only when pCR was achieved and severe hematotoxicity did not occur; all other joint outcome combinations were coded as 0. Because the utility score represents a continuous benefit–toxicity measure rather than a calibrated probability, it was mapped to the predicted probability of the favorable composite outcome before decision curve analysis. Specifically, a univariable logistic mapping model was fitted exclusively in the training cohort using utility scores calculated from training-cohort out-of-fold predictions, with the observed favorable composite outcome as the binary dependent variable. The resulting intercept and slope were fixed and applied unchanged to the internal and external validation cohorts to obtain predicted favorable-outcome probabilities. No recalibration or parameter updating was performed in either validation cohort. Decision curve analysis was subsequently performed using these predicted probabilities, with the observed favorable composite outcome as the clinical endpoint, and net benefit was compared with the treat-all and treat-none strategies across threshold probabilities.

As a complementary representation of the two multitask predictions, the predicted probabilities of pCR and severe hematotoxicity were jointly displayed in a two-dimensional benefit–risk space. Predicted severe hematotoxicity probability was plotted on the *x*-axis and predicted pCR probability on the *y*-axis. The task-specific classification thresholds derived from training-cohort out-of-fold predictions using the Youden index were used to divide the prediction space into four profiles: high predicted pCR/low predicted hematotoxicity, high predicted pCR/high predicted hematotoxicity, low predicted pCR/low predicted hematotoxicity, and low predicted pCR/high predicted hematotoxicity. The same fixed thresholds were applied unchanged to the internal and external validation cohorts.

The clinical relevance of the utility-based stratification was evaluated by comparing observed pCR rates, severe hematotoxicity rates, favorable composite-outcome rates, and secondary prognostic outcomes across benefit groups. PFS and OS were analyzed using Kaplan–Meier curves and compared using the log-rank test.

### 2.6. Statistical Analysis

All statistical analyses and model development were performed using Python version 3.11 (Python Software Foundation, Wilmington, DE, USA). Continuous variables are presented as mean ± standard deviation or median with interquartile range, as appropriate, and categorical variables as frequencies and percentages. Comparisons across the training, internal validation, and external validation cohorts were performed using one-way analysis of variance or the Kruskal–Wallis test for continuous variables and the chi-square test or Fisher’s exact test for categorical variables, as appropriate. Pairwise standardized mean differences (SMDs) were additionally calculated to quantify the magnitude of baseline differences between cohorts. Differences in categorical outcomes across utility-based benefit groups were assessed using the chi-square test or Fisher’s exact test, as appropriate. Model discrimination was evaluated using the area under the receiver operating characteristic curve (AUC), and differences in AUCs between models were compared using the DeLong test. PFS and OS were estimated using the Kaplan–Meier method, and survival differences between groups were compared using the log-rank test. All statistical tests were two-sided, and *p* < 0.05 was considered statistically significant.

## 3. Results

### 3.1. Patient Characteristics

A total of 2060 patients were included, comprising a training cohort (*n* = 1406), an internal validation cohort (*n* = 351), and an external validation cohort (*n* = 303). Across the three cohorts, the mean age was approximately 50 years, and the mean BMI was approximately 24 kg/m^2^. Most patients presented with stage II–III disease, and tumors measuring 20–50 mm were the most common. Planned anthracycline exposure was recorded in approximately two-thirds of patients, whereas planned immunotherapy exposure was relatively uncommon. Axillary lymph node positivity was also frequent across the cohorts. Overall, baseline clinical, pathological, imaging, and electrocardiographic characteristics were comparable among the training, internal validation, and external validation cohorts, with no statistically significant differences observed across the examined variables (all *p* > 0.05, Table 1). Pairwise standardized mean differences were generally small, and all SMDs for baseline clinical characteristics were below 0.20 (Supplementary Table S4).

Laboratory parameters, including liver function, renal function, hematological indices, and coagulation markers, were also comparable across the three cohorts, with no statistically significant differences observed for the examined laboratory variables (all *p* > 0.05, Table 2). Pairwise standardized mean differences were generally small, and all SMDs for baseline laboratory characteristics were below 0.20 (Supplementary Table S5).

### 3.2. Treatment Outcomes and Severe Hematotoxicity

**Figure 2:**
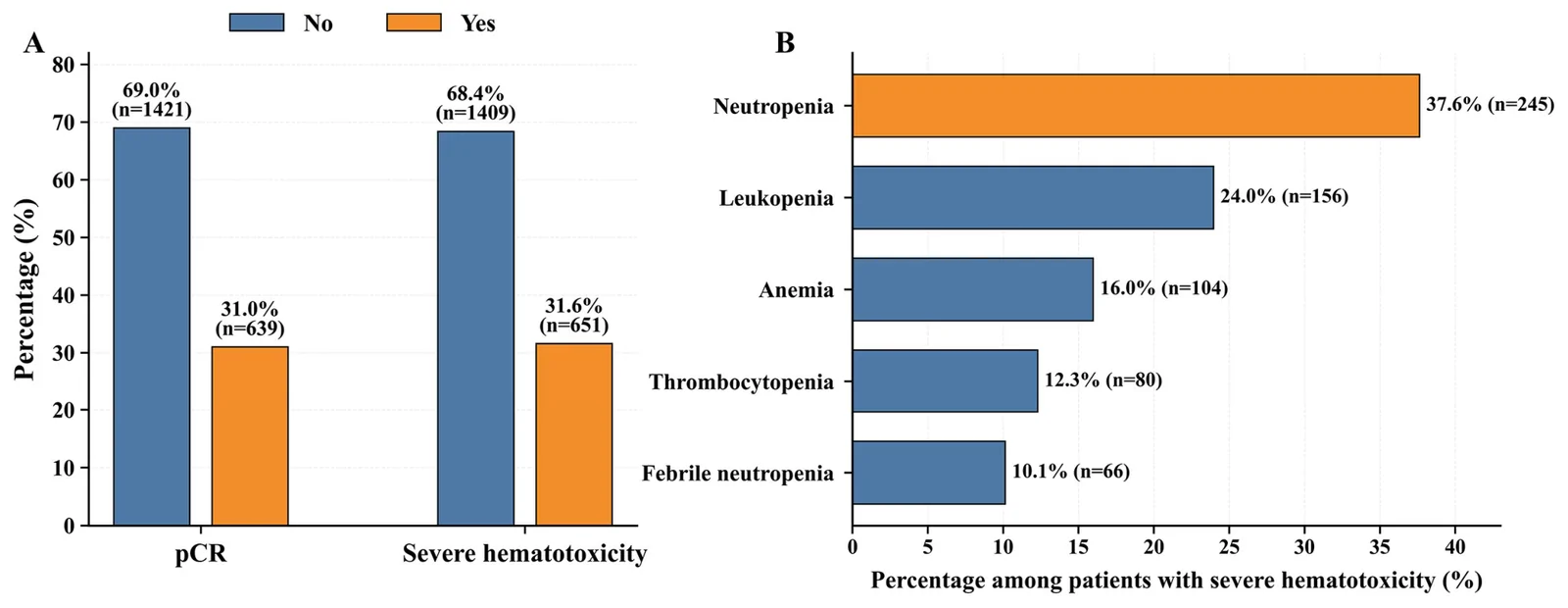
Treatment outcomes and severe hematotoxicity in the study population. (**A**) Distribution of pCR and severe hematotoxicity status in the overall study population. (**B**) Distribution of recorded hematotoxicity types among patients with severe hematotoxicity. pCR, pathological complete response.

Among the 2060 patients included in the analysis, 639 (31.0%) achieved pCR, whereas 1421 (69.0%) did not. The pCR rates were 31.0% (436/1406), 31.1% (109/351), and 31.0% (94/303) in the training, internal validation, and external validation cohorts, respectively. Severe hematotoxicity occurred in 651 patients (31.6%) overall, whereas 1409 patients (68.4%) did not develop severe hematotoxicity (Figure 2A). The corresponding severe hematotoxicity rates were 32.6% (458/1406), 30.5% (107/351), and 28.4% (86/303) in the training, internal validation, and external validation cohorts, respectively. Among the 651 patients with severe hematotoxicity, neutropenia was the most frequently recorded type, accounting for 37.6% (*n* = 245), followed by leukopenia (24.0%, *n* = 156), anemia (16.0%, *n* = 104), thrombocytopenia (12.3%, *n* = 80), and febrile neutropenia (10.1%, *n* = 66) (Figure 2B).

### 3.3. Model Performance for pCR and Severe Hematotoxicity Prediction

**Figure 3:**
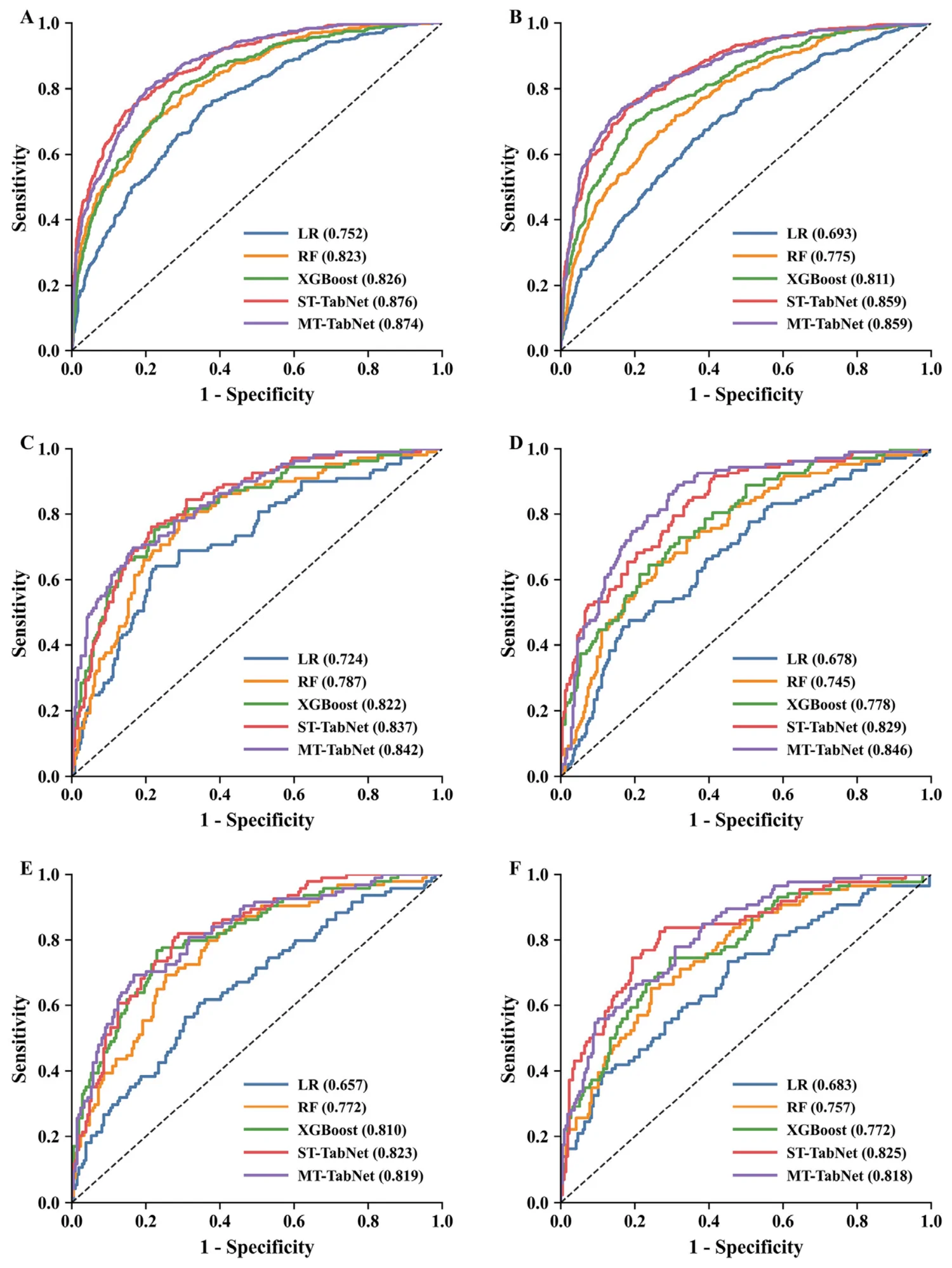
ROC curves of different prediction models across the study cohorts. (**A**) ROC curves for pCR prediction in the training cohort. (**B**) ROC curves for severe hematotoxicity prediction in the training cohort. (**C**) ROC curves for pCR prediction in the internal validation cohort. (**D**) ROC curves for severe hematotoxicity prediction in the internal validation cohort. (**E**) ROC curves for pCR prediction in the external validation cohort. (**F**) ROC curves for severe hematotoxicity prediction in the external validation cohort. LR, logistic regression; RF, random forest; XGBoost, extreme gradient boosting; ST-TabNet, single-task TabNet; MT-TabNet, multitask TabNet.

For pCR prediction, the MT-TabNet model achieved AUCs of 0.874 (95% CI, 0.849–0.903), 0.842 (95% CI, 0.787–0.889), and 0.819 (95% CI, 0.744–0.876) in the training, internal validation, and external validation cohorts, respectively (Figure 3A,C,E). Across the three cohorts, sensitivity ranged from 0.746 to 0.787, specificity from 0.747 to 0.796, and Brier scores from 0.145 to 0.169, with corresponding PPVs of 0.570–0.634 and NPVs of 0.867–0.893 (Table 3). Detailed confidence intervals and calibration metrics are provided in Supplementary Table S6.

**Figure 4:**
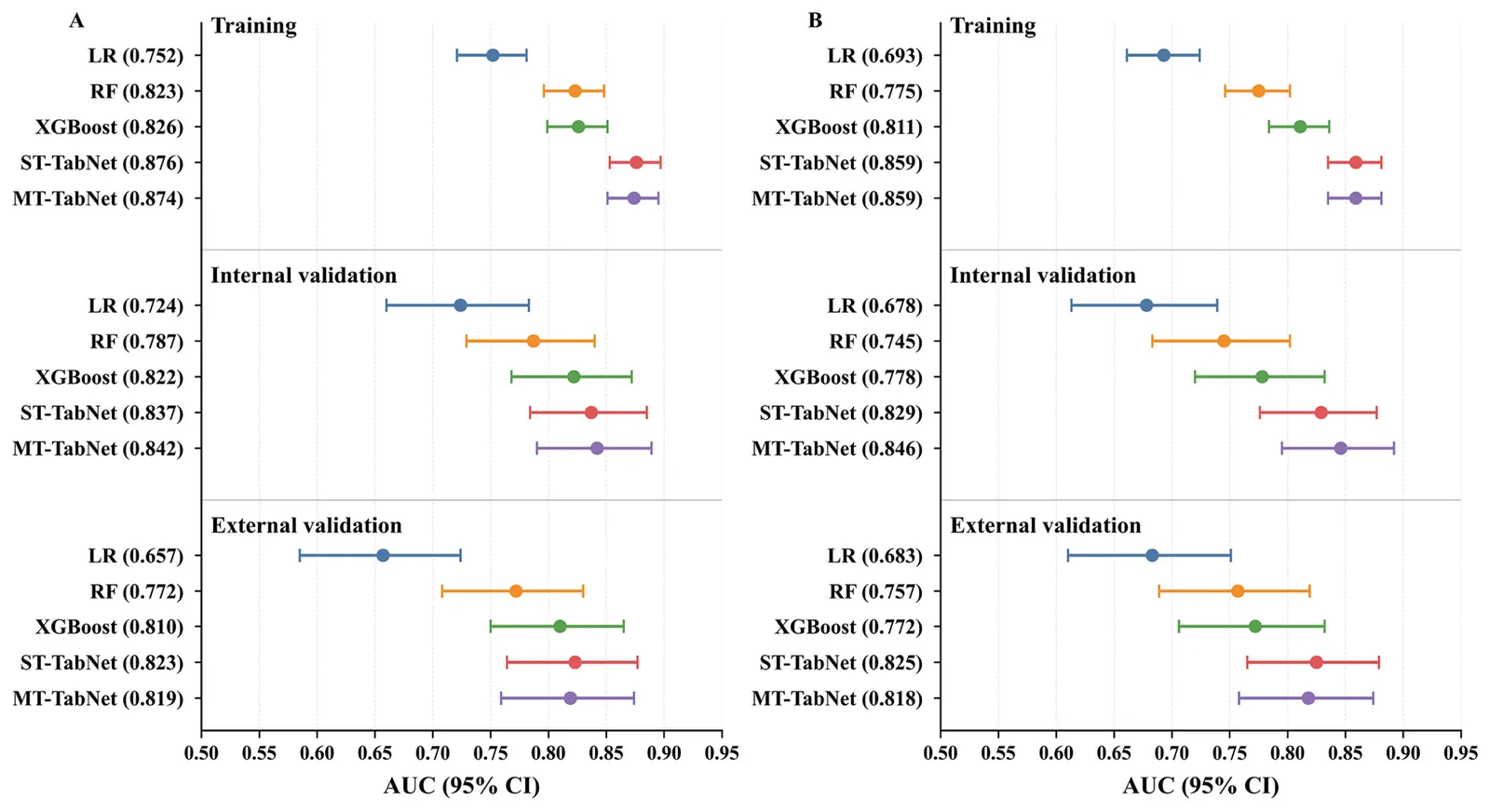
Comparison of model discrimination across prediction tasks and study cohorts. (**A**) AUCs and corresponding 95% confidence intervals for pCR prediction across the training, internal validation, and external validation cohorts. (**B**) AUCs and corresponding 95% confidence intervals for severe hematotoxicity prediction across the three cohorts. Abbreviations: CI, confidence interval; LR, logistic regression; RF, random forest; XGBoost, extreme gradient boosting; ST-TabNet, single-task TabNet; MT-TabNet, multitask TabNet.

Compared with conventional models, MT-TabNet showed higher AUCs than LR, RF, and XGBoost for pCR prediction across all three cohorts (Figure 4A). DeLong testing showed that the AUC of MT-TabNet was significantly higher than those of LR, RF, and XGBoost in the training cohort (all *p* < 0.001). In the internal validation cohort, the difference remained significant compared with LR (*p* = 0.003), whereas differences compared with RF, XGBoost, and ST-TabNet were not statistically significant. Similarly, in the external validation cohort, MT-TabNet significantly outperformed LR (*p* < 0.001), whereas no significant differences were observed compared with RF, XGBoost, or ST-TabNet (Supplementary Table S7).

For severe hematotoxicity prediction, MT-TabNet achieved AUCs of 0.859 (95% CI, 0.845–0.888), 0.846 (95% CI, 0.797–0.896), and 0.818 (95% CI, 0.757–0.871) in the training, internal validation, and external validation cohorts, respectively (Figure 3B,D,F). Sensitivity ranged from 0.724 to 0.776 and specificity from 0.774 to 0.788, while Brier scores ranged from 0.143 to 0.169 across cohorts (Table 3). MT-TabNet showed higher AUCs than LR, RF, and XGBoost across all three cohorts (Figure 4B). DeLong testing demonstrated significant differences compared with LR, RF, and XGBoost in the training cohort (all *p* < 0.001) and in the internal validation cohort (*p* < 0.001, *p* = 0.009, and *p* = 0.041, respectively). In the external validation cohort, MT-TabNet remained significantly better than LR (*p* = 0.005), whereas differences compared with RF, XGBoost, and ST-TabNet were not statistically significant (Supplementary Table S8).

Task-level comparisons between MT-TabNet and ST-TabNet showed that multitask learning did not uniformly improve discrimination across all evaluation settings. In repeated cross-validation, changes in AUC were small for both pCR and severe hematotoxicity prediction (both ΔAUC = +0.003), with confidence intervals including zero. In the internal validation cohort, severe hematotoxicity prediction showed the clearest evidence of positive transfer, with a ΔAUC of +0.017 (95% CI, 0.001–0.033) accompanied by a reduction in Brier score (ΔBrier = +0.006, 95% CI, 0.000–0.012). In contrast, the corresponding differences for pCR prediction and for both tasks in the external validation cohort were small, with confidence intervals spanning zero (Supplementary Table S9). Overall, MT-TabNet maintained discriminative performance comparable to ST-TabNet while providing joint estimation of pathological response and severe hematotoxicity within a unified multitask framework.

### 3.4. Further Evaluation of the MT-TabNet Model

**Figure 5:**
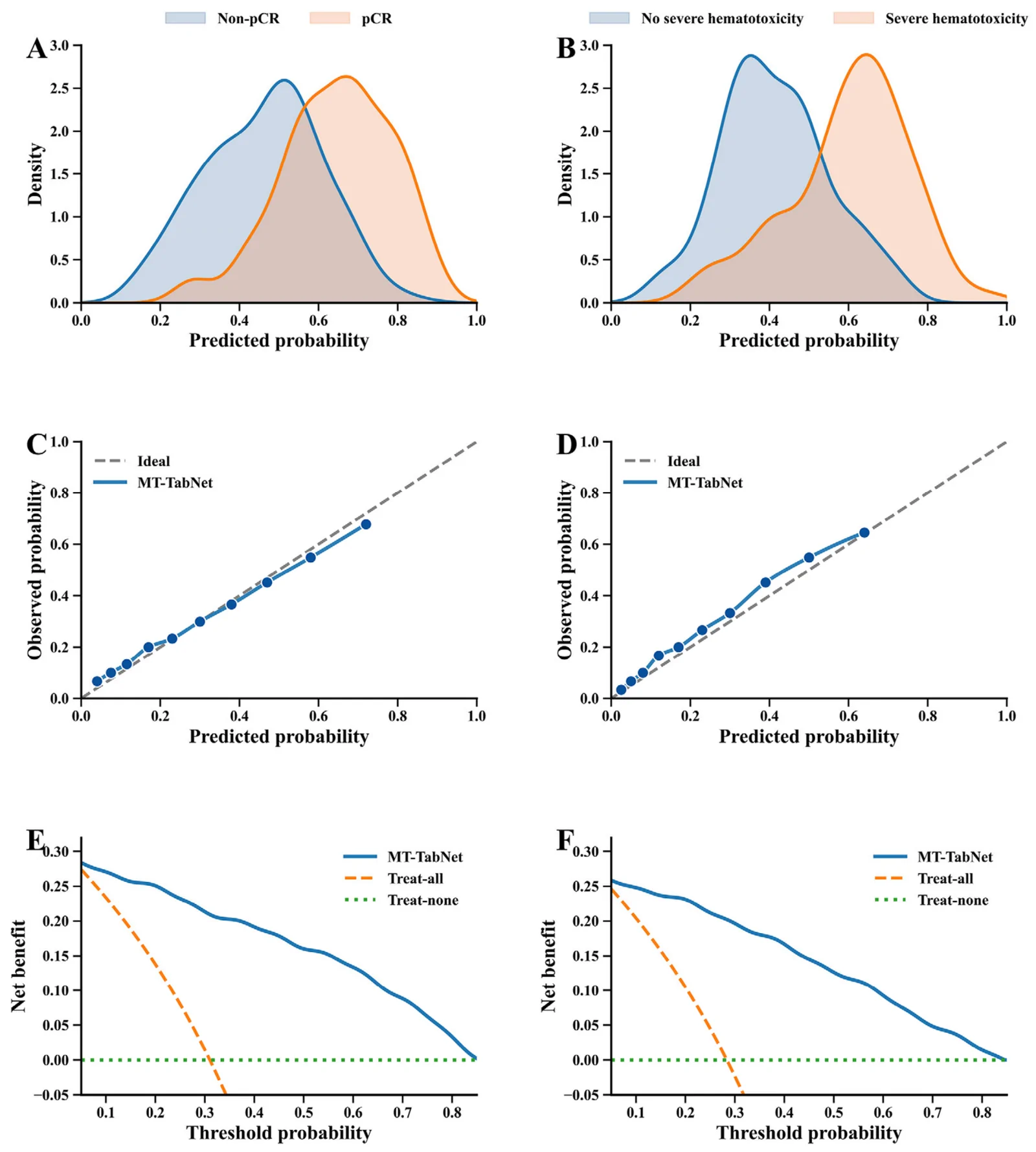
Performance evaluation of the MT-TabNet model in the external validation cohort. (**A**) Distribution of predicted probabilities for pCR prediction. (**B**) Distribution of predicted probabilities for severe hematotoxicity prediction. (**C**) Calibration curve for pCR prediction. (**D**) Calibration curve for severe hematotoxicity prediction. (**E**) Decision curve analysis for pCR prediction. (**F**) Decision curve analysis for severe hematotoxicity prediction. Abbreviations: pCR, pathological complete response; MT-TabNet, multitask TabNet.

In the external validation cohort, predicted probability distributions, calibration, and decision curve analysis were used to further evaluate the MT-TabNet model. For pCR prediction, patients who achieved pCR had generally higher predicted probabilities than those who did not, with clear separation between the two distributions (Figure 5A). Similarly, patients who developed severe hematotoxicity had higher predicted probabilities than those without severe hematotoxicity, although some overlap between the distributions remained (Figure 5B).

Calibration analysis showed generally good agreement between predicted and observed probabilities for both prediction tasks, with only modest deviations from the ideal calibration line across the probability range (Figure 5C,D). Calibration intercepts, calibration slopes, and their corresponding confidence intervals are provided in Supplementary Table S6. Decision curve analysis showed that MT-TabNet provided greater net benefit than the treat-all and treat-none strategies across a range of clinically relevant threshold probabilities for both pCR and severe hematotoxicity predictions (Figure 5E,F).

### 3.5. Model Interpretability and Feature Analysis

**Figure 6:**
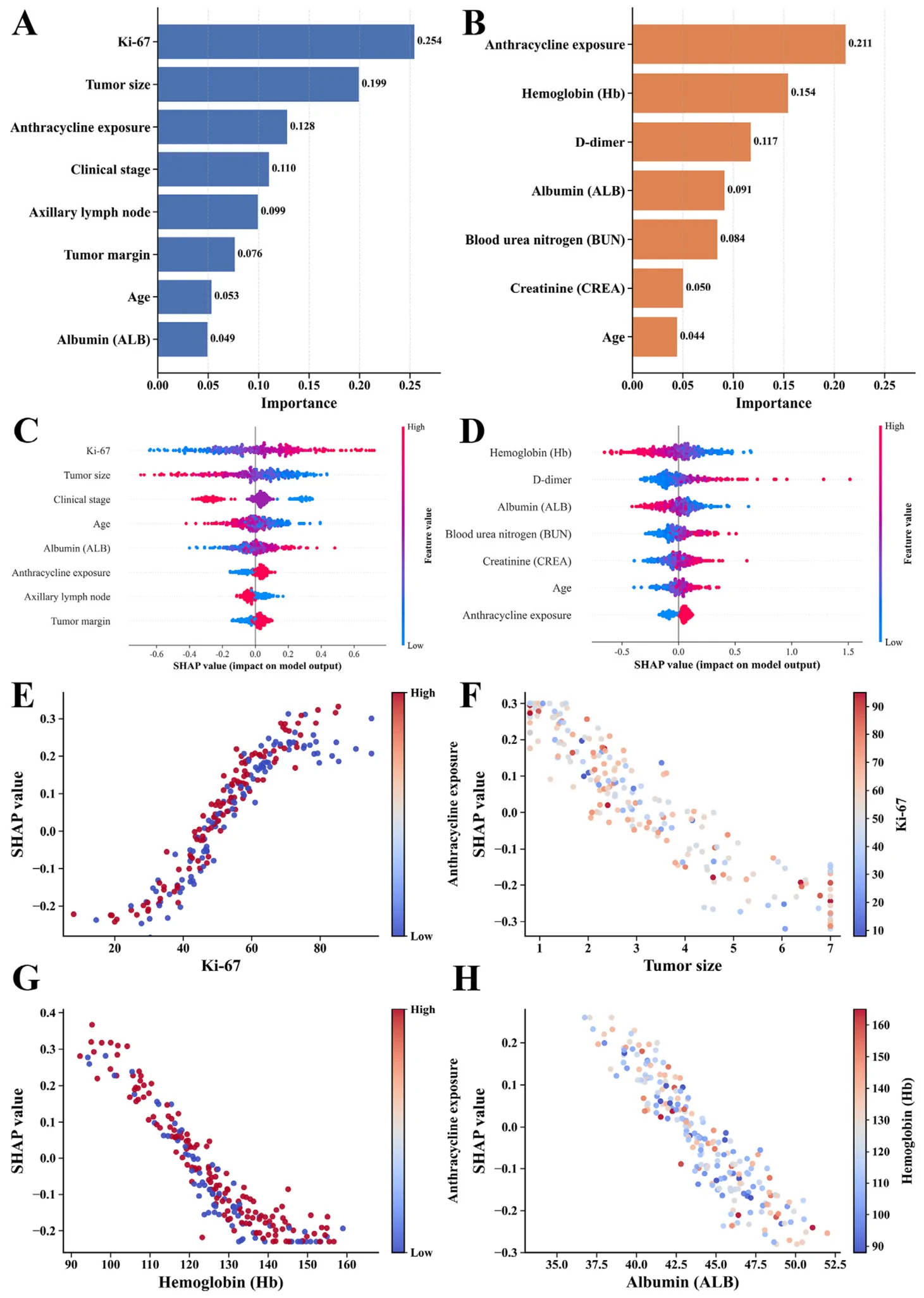
Interpretability analysis of the multitask TabNet model. (**A**) Feature importance ranking for pCR prediction. (**B**) Feature importance ranking for severe hematotoxicity prediction. (**C**) SHAP summary plot for pCR prediction. (**D**) SHAP summary plot for severe hematotoxicity prediction. (**E**) SHAP dependence plot of Ki-67 for pCR prediction. (**F**) SHAP dependence plot of tumor size for pCR prediction. (**G**) SHAP dependence plot of hemoglobin for severe hematotoxicity prediction. (**H**) SHAP dependence plot of albumin for severe hematotoxicity prediction. SHAP, SHapley Additive exPlanations; Hb, hemoglobin; ALB, albumin; BUN, blood urea nitrogen; CREA, creatinine.

Feature importance analysis identified distinct predictor patterns for the two tasks. For pCR prediction, Ki-67 was the most important variable, followed by tumor size, planned anthracycline exposure, clinical stage, and axillary lymph node status (Figure 6A). For severe hematotoxicity prediction, planned anthracycline exposure showed the highest importance, followed by hemoglobin, D-dimer, albumin, blood urea nitrogen, creatinine, and age (Figure 6B).

SHAP summary plots further characterized the direction and distribution of feature contributions. For pCR prediction, higher Ki-67 values were associated with positive SHAP values, whereas larger tumor size and more advanced clinical stage were associated with negative SHAP values (Figure 6C). For severe hematotoxicity prediction, lower hemoglobin levels and higher D-dimer values were associated with higher predicted risk, whereas higher albumin levels were associated with lower model output (Figure 6D).

SHAP dependence plots demonstrated nonlinear associations between key predictors and model outputs. For pCR prediction, Ki-67 showed a nonlinear positive association with SHAP values, whereas tumor size showed a decreasing trend in SHAP values with increasing tumor burden (Figure 6E,F). For severe hematotoxicity prediction, hemoglobin and albumin both showed inverse associations with SHAP values, suggesting that lower baseline hematologic and nutritional status contributed to higher predicted hematotoxicity risk (Figure 6G,H).

These interpretability analyses indicated that pCR prediction was mainly driven by tumor-related variables, including Ki-67, tumor size, clinical stage, and axillary lymph node status, whereas severe hematotoxicity prediction was mainly driven by treatment exposure and host-related laboratory indicators, including anthracycline exposure, hemoglobin, D-dimer, albumin, renal function markers, and age. Shared variables, such as anthracycline exposure, age, and albumin, contributed to both tasks but showed different relative importance and contribution patterns.

### 3.6. Feature Ablation Analysis Across Multitask Outcomes

**Figure 7:**
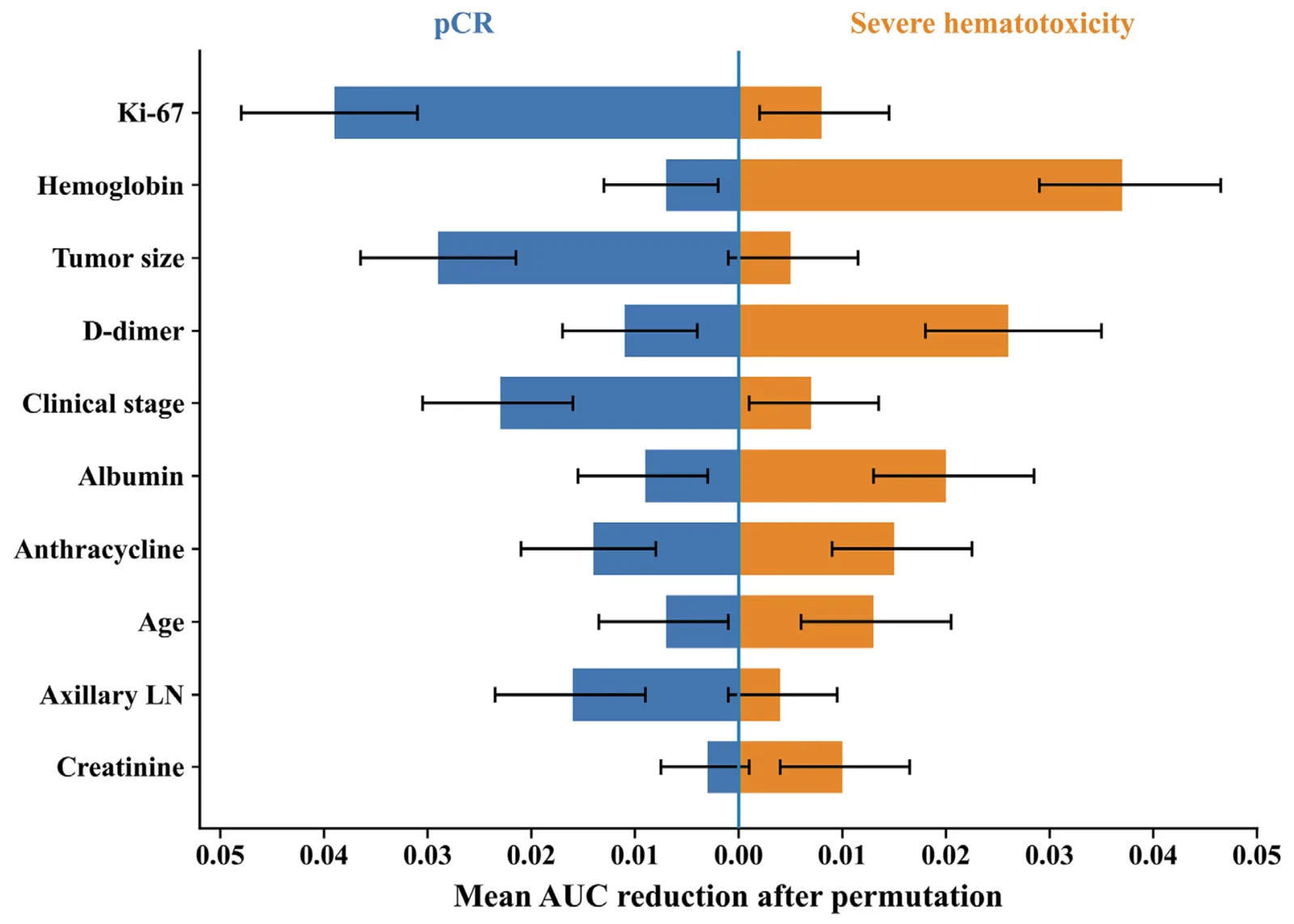
Feature ablation analysis across multitask outcomes. Permutation-based feature ablation analysis showing the mean decrease in AUC and corresponding 95% confidence intervals based on 100 permutation repetitions for individual predictors in pCR prediction and severe hematotoxicity prediction. AUC, area under the receiver operating characteristic curve; pCR, pathological complete response; LN, lymph node.

Permutation-based feature ablation analysis further quantified the contribution of individual variables to each prediction task (Figure 7). Each predictor was permuted 100 times, and its contribution was summarized by the mean reduction in AUC with the corresponding 95% confidence interval. For pCR prediction, perturbation of Ki-67 produced the largest decrease in AUC, followed by tumor size, clinical stage, anthracycline exposure, and axillary lymph node status. In contrast, severe hematotoxicity prediction was more strongly influenced by host-related laboratory indicators. Hemoglobin showed the largest AUC reduction after perturbation, followed by D-dimer, albumin, renal function markers, and age.

Several variables contributed to both tasks but differed in magnitude. Anthracycline exposure showed measurable effects on both pCR and severe hematotoxicity prediction, whereas albumin and age had weaker but consistent cross-task contributions. These findings support the multitask framework by showing that the two outcomes shared some predictive information while retaining distinct task-specific feature patterns.

### 3.7. Clinical Utility and Decision Stratification

**Figure 8:**
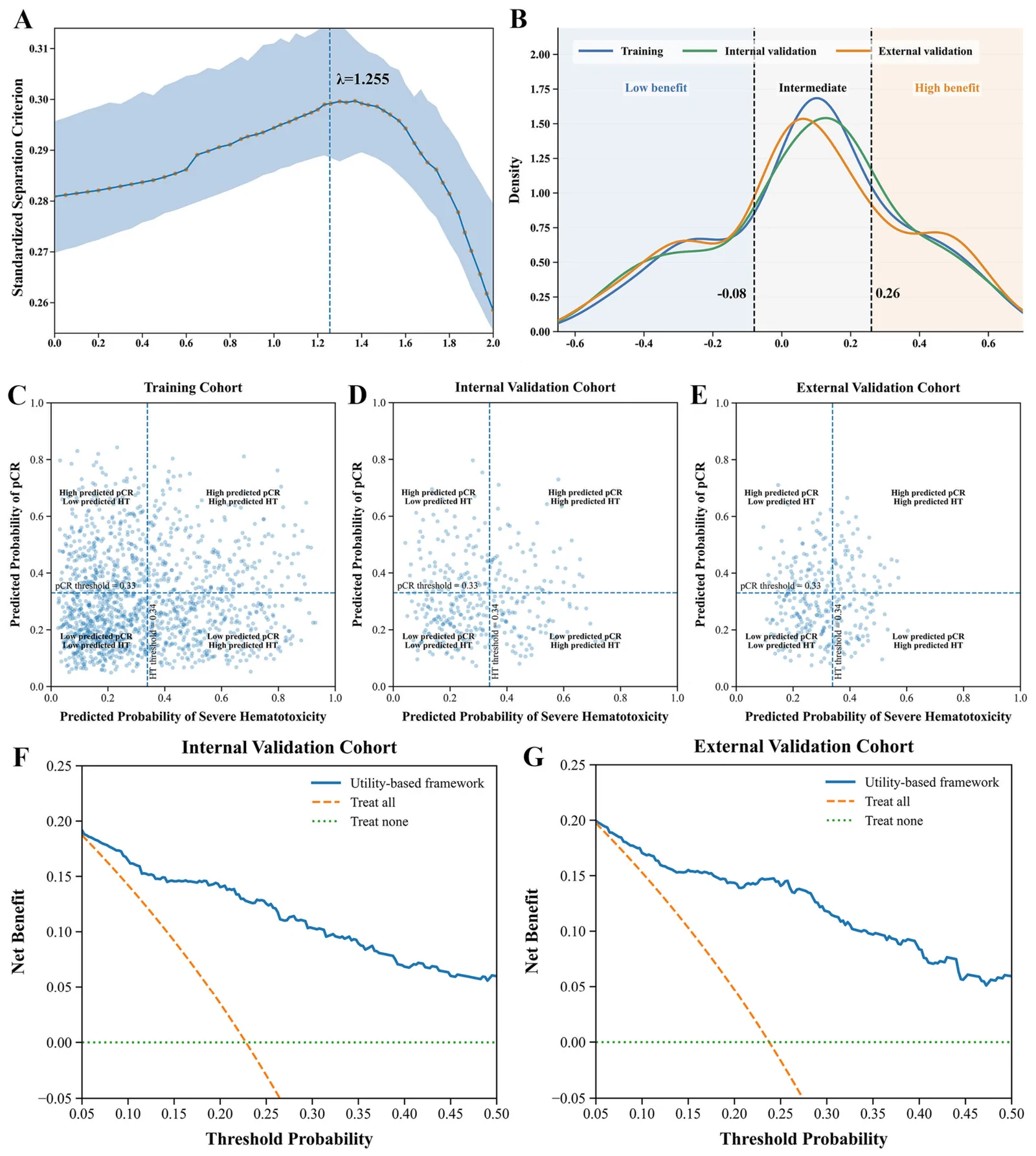
Utility-based clinical benefit stratification. (**A**) Standardized separation criterion across λ values. (**B**) Utility-score distributions and benefit-group stratification across the study cohorts. (**C**) Two-dimensional benefit–risk matrix in the training cohort. (**D**) Two-dimensional benefit–risk matrix in the internal validation cohort. (**E**) Two-dimensional benefit–risk matrix in the external validation cohort. (**F**) Decision curve analysis in the internal validation cohort. (**G**) Decision curve analysis in the external validation cohort. pCR, pathological complete response; HT, severe hematotoxicity.

In the training cohort, the standardized separation criterion reached its maximum at λ = 1.255, with a relatively broad peak around the selected value (Figure 8A). The bootstrap confidence band showed substantial overlap around the peak region. At λ = 1.255, the 25th and 75th percentiles of the training-cohort utility score were −0.08 and 0.26, respectively. These two cutoffs were fixed and applied to the internal and external validation cohorts to classify patients into low-, intermediate-, and high-benefit groups (Figure 8B).

The paired predicted probabilities of pCR and severe hematotoxicity were further visualized in a two-dimensional benefit–risk space across the training, internal validation, and external validation cohorts (Figure 8C–E). Predicted severe hematotoxicity probability was plotted on the *x*-axis and predicted pCR probability on the *y*-axis. Using the fixed task-specific classification thresholds derived from the training-cohort out-of-fold predictions, the prediction space was divided into four profiles: high predicted pCR/low predicted hematotoxicity, high predicted pCR/high predicted hematotoxicity, low predicted pCR/low predicted hematotoxicity, and low predicted pCR/high predicted hematotoxicity. All four prediction profiles were represented across the three cohorts, with broadly similar overall distribution patterns.

The utility-based stratification showed consistent outcome gradients across the three cohorts (Table 4). In the training cohort, the pCR rate increased from 17.3% in the low-benefit group to 44.9% in the high-benefit group, whereas the rate of severe hematotoxicity decreased from 44.0% to 22.2%. Similar patterns were observed in the internal validation cohort, with pCR rates increasing from 16.7% to 45.3% and severe hematotoxicity rates decreasing from 46.4% to 17.4%. In the external validation cohort, the pCR rate increased from 16.4% in the low-benefit group to 46.1% in the high-benefit group, while the rate of severe hematotoxicity decreased from 45.2% to 15.8%. Differences across the three benefit groups were statistically significant for both outcomes in all three cohorts (all *p* < 0.001). Favorable-outcome rates, defined as pCR without severe hematotoxicity, also increased progressively from the low- to the high-benefit group across the three cohorts.

Decision curve analyses based on the predicted probability of the favorable composite outcome showed that the utility-based framework provided greater net benefit than the treat-all and treat-none strategies across a clinically relevant range of threshold probabilities in both the internal and external validation cohorts (Figure 8F,G).

Sensitivity analyses using alternative λ values of 0.5, 1.0, 1.5, and 2.0 showed similar overall stratification patterns (Supplementary Table S10). As λ increased, the difference in pCR rates between the high- and low-benefit groups decreased, whereas the difference in severe hematotoxicity rates between the low- and high-benefit groups increased. The difference in favorable-outcome rates between the high- and low-benefit groups remained relatively stable across the evaluated λ values, ranging from 27.0% to 30.9%.

### 3.8. Survival Analysis

**Figure 9:**
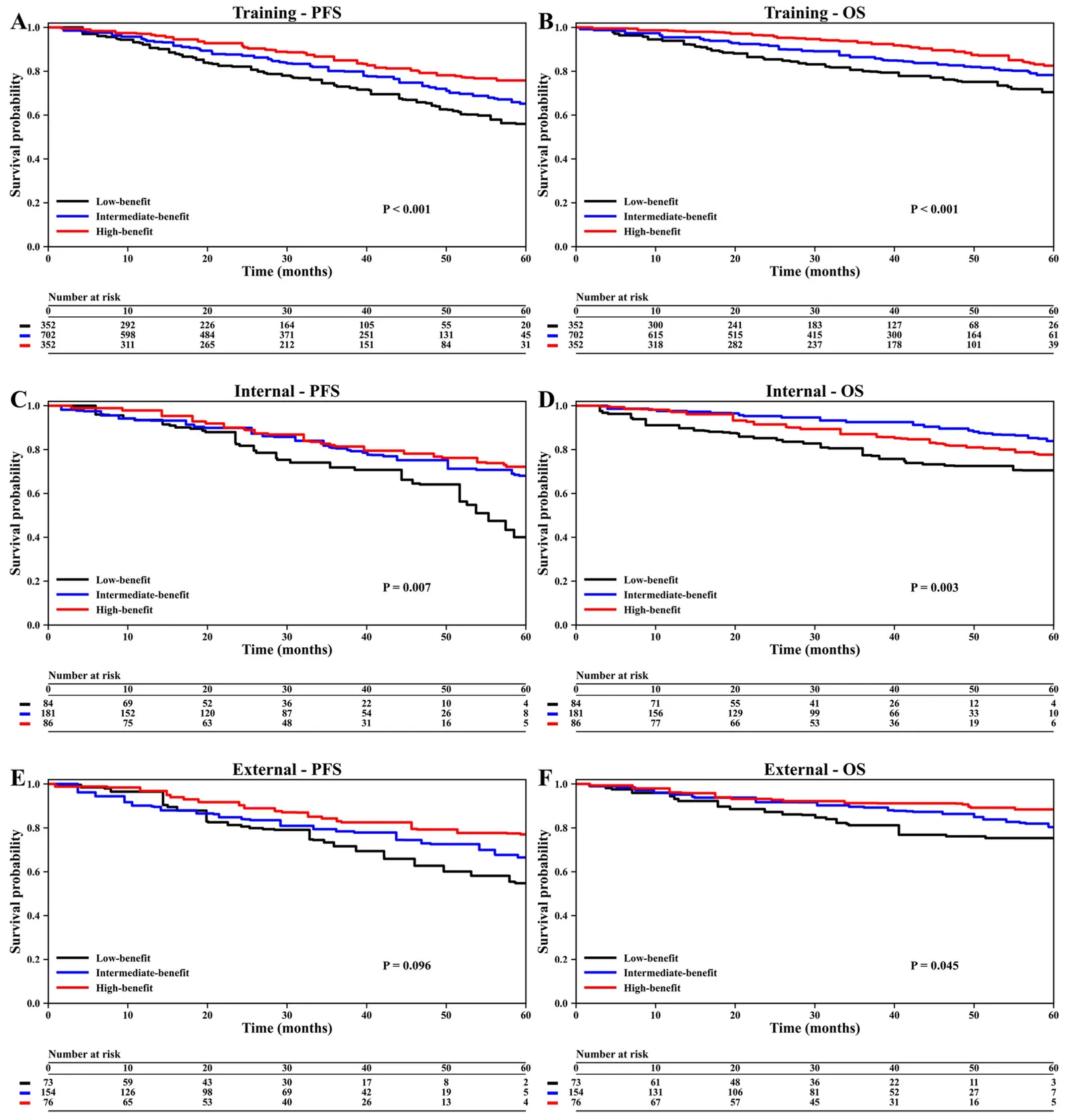
Survival outcomes according to utility-based benefit groups. (**A**) PFS in the training cohort. (**B**) OS in the training cohort. (**C**) PFS in the internal validation cohort. (**D**) OS in the internal validation cohort. (**E**) PFS in the external validation cohort. (**F**) OS in the external validation cohort. Numbers at risk are shown below each panel. PFS, progression-free survival; OS, overall survival.

Patients in each cohort were stratified into low-, intermediate-, and high-benefit groups according to the utility-based framework. In the training cohort, significant differences were observed among the three benefit groups for both PFS and OS (both *p* < 0.001; Figure 9A,B), with the high-benefit group showing the most favorable survival outcomes. Similar findings were observed in the internal validation cohort, where significant differences were identified for both PFS (*p* = 0.007; Figure 9C) and OS (*p* = 0.003; Figure 9D). In the external validation cohort, PFS showed a similar trend across the benefit groups, although the difference did not reach statistical significance (*p* = 0.096; Figure 9E). OS differed significantly among the three groups (*p* = 0.045; Figure 9F), with the high-benefit group showing the most favorable overall survival. Overall, the utility-based benefit groups were associated with differences in long-term outcomes, although PFS separation was not statistically significant in the external validation cohort.

## 4. Discussion

In this study, we developed and validated a TabNet-based multitask learning framework for patients with TNBC receiving platinum-based neoadjuvant therapy to simultaneously estimate the probability of pCR and the risk of severe hematotoxicity. The MT-TabNet model maintained good discriminative performance across the training, internal validation, and external validation cohorts, with generally acceptable calibration and clinical net benefit. Interpretability analyses showed distinct predictor patterns for the two tasks: pCR prediction was predominantly associated with tumor-related characteristics, whereas severe hematotoxicity prediction was more strongly influenced by planned treatment exposure and host-related laboratory indicators. The utility-based framework integrating the two model outputs further stratified patients into low-, intermediate-, and high-benefit groups, with progressively higher pCR rates and lower severe hematotoxicity rates across the three cohorts. Survival analyses also showed differences among benefit groups, with significant PFS and OS differences in the training and internal validation cohorts and a significant OS difference in the external validation cohort, although the difference in PFS in the external validation cohort did not reach statistical significance.

The MT-TabNet model showed predictive performance generally comparable to that of ST-TabNet for both pCR and severe hematotoxicity. The methodological rationale for multitask learning in this setting lies in its ability to jointly model two clinically related but distinct outcomes within a unified representation-learning framework. In contrast to two independently trained single-task models, in which each task develops its own feature representation and optimizes its own objective without direct information exchange, MT-TabNet uses a shared representation that is jointly updated by the learning signals from both tasks, while task-specific output layers preserve outcome-specific predictive patterns [24,25,26,27]. This architecture allows information that is potentially relevant to both efficacy and toxicity to contribute to a common latent representation without requiring the two outcomes to share identical determinants.

This distinction also affects how the model outputs can be interpreted and subsequently analyzed. Two independently developed single-task models can separately estimate the probability of pCR and the probability of severe hematotoxicity, but these predictions arise from independent modeling processes and therefore require subsequent integration outside the models when the efficacy–toxicity trade-off is considered. In contrast, MT-TabNet simultaneously generates paired individualized estimates of pathological response and severe hematotoxicity for the same patient using the same pretreatment information within a unified shared modeling framework. These two outputs can still be evaluated separately in terms of discrimination, calibration, and clinical utility, while also providing a coherent basis for examining their joint distribution and for subsequent benefit–hematotoxicity stratification. This is particularly relevant in platinum-based neoadjuvant therapy, where treatment assessment requires simultaneous consideration of expected pathological benefit and severe hematotoxicity risk rather than either outcome in isolation [28]. Accordingly, the principal advantage of the multitask framework in the present study should be interpreted as the joint utilization of shared predictive information, simultaneous estimation of two complementary clinical outcomes, and provision of a unified basis for patient-level benefit–toxicity assessment. This advantage is conceptually distinct from improvement in task-specific discrimination, and the relatively small differences observed between MT-TabNet and ST-TabNet indicate that multitask learning should not be expected to universally increase the AUC of each individual task.

Model interpretability analyses further supported the clinical plausibility of joint modeling. The most important variables for pCR prediction included Ki-67, tumor size, clinical stage, and axillary lymph node status, which mainly reflect tumor proliferative activity, tumor burden, and disease extent [29,30]. Higher Ki-67 was associated with a higher predicted probability of pCR, consistent with the clinical understanding that highly proliferative tumors may be more sensitive to cytotoxic therapy [31]. In contrast, larger tumor size and more advanced clinical stage were associated with a lower predicted probability of pathological complete response [32]. Severe hematotoxicity prediction was more strongly associated with planned anthracycline exposure and host-related factors, including hemoglobin, D-dimer, albumin, blood urea nitrogen, creatinine, and age [33,34]. These variables may reflect baseline hematopoietic reserve, nutritional and inflammatory status, renal function, and differences in the planned treatment regimen [35]. In other words, pCR prediction was more closely related to tumor biology and disease burden, whereas severe hematotoxicity prediction was more closely associated with host tolerance and planned anthracycline exposure.

Some variables contributed to both tasks but differed in direction and magnitude. Planned anthracycline exposure contributed to both pCR and severe hematotoxicity prediction, indicating that the characteristics of the planned treatment regimen may be relevant to both treatment response and toxicity risk [36]. Age and albumin also showed cross-task contributions, indicating that overall physiological reserve and nutritional and inflammatory status may influence both treatment response and treatment tolerance [37,38]. The consistency between feature ablation analysis and SHAP-based interpretation further suggests that the model did not rely on a single incidental variable but captured multidimensional predictive signals consistent with clinical knowledge [39].

On this basis, the utility framework was used to integrate the predicted probabilities of pCR and severe hematotoxicity into a single measure of the expected efficacy–toxicity balance. This formulation reflects the clinical setting in which a higher probability of pathological response represents greater expected treatment benefit, whereas a higher probability of severe hematotoxicity represents greater treatment-related burden. Considering these two dimensions jointly may therefore provide information that is not captured by either prediction alone, particularly when predicted efficacy and toxicity are simultaneously substantial. The utility score provides a transparent summary in which expected pathological benefit increases the overall utility, whereas expected severe hematotoxicity reduces it [40]. Across the three cohorts, the resulting stratification identified groups with progressively higher pCR rates and lower severe hematotoxicity rates, supporting the potential clinical relevance of this integrated assessment and providing a basis for future risk-adapted monitoring and supportive-care strategies [41,42].

Within this framework, λ determines the relative weight assigned to severe hematotoxicity when pathological benefit and toxicity are considered jointly. The reference value of λ = 1.255 obtained in the present study means that a 10-percentage-point increase in the predicted probability of severe hematotoxicity decreases the utility score by 0.1255, which would be mathematically offset by an approximately 12.6-percentage-point increase in the predicted probability of pCR. Thus, larger λ values place greater emphasis on avoiding severe hematotoxicity, whereas smaller λ values place relatively greater emphasis on achieving pathological response. This relationship should not be interpreted as a clinically validated fixed exchange rate between pathological benefit and toxicity, but rather as the clinical interpretation of the relative weighting represented by λ.

The reference λ was estimated using the two concordant joint-outcome states, namely pCR without severe hematotoxicity and non-pCR with severe hematotoxicity. These outcomes represent clinically distinct states at opposite ends of the efficacy–toxicity spectrum, with pathological benefit and toxicity status being concordantly favorable or unfavorable, respectively. Separation between these reference states was quantified using a standardized criterion that incorporated both the difference in mean utility scores and the within-state dispersion of utility values. By accounting for both between-state separation and within-state variability, this criterion provided a more comprehensive measure of how clearly a given λ distinguished favorable from unfavorable efficacy–toxicity profiles. Importantly, these two outcome states were used only to estimate the reference λ. Once λ was determined, it was applied to all patients, regardless of their observed joint-outcome category, and the subsequent benefit-stratification cutoffs were derived from the utility-score distribution of the entire training cohort.

The clinically appropriate value of λ may nevertheless vary substantially according to individual patient preferences. Patients who place greater importance on avoiding severe hematotoxicity may favor a larger λ, whereas those who are more willing to accept toxicity in exchange for a higher probability of pathological response may favor a smaller λ. Therefore, the λ = 1.255 derived in the present study should be regarded as a cohort-level reference weight rather than a universally optimal patient-level preference. The broadly consistent stratification patterns observed across alternative λ values in the sensitivity analyses suggest that the overall framework was not dependent on a single weighting scenario, while also illustrating its potential flexibility under different relative preferences for efficacy and toxicity.

Survival analysis further supported the potential clinical relevance of the stratification framework. Although the utility score was constructed from short-term pathological benefit and severe hematotoxicity risk, significant differences in both PFS and OS were observed across benefit groups in the training and internal validation cohorts. In the external validation cohort, OS differed significantly among the three groups, whereas PFS showed a similar trend but did not reach statistical significance. This suggests that the short-term efficacy–toxicity profile may be associated with long-term prognosis, although the prognostic value of the stratification requires further validation. In TNBC neoadjuvant therapy, pCR is closely associated with favorable long-term outcomes [43,44], whereas severe hematotoxicity may affect treatment tolerance and overall clinical condition [45]. The present framework extends the role of predictive modeling from estimating isolated outcomes to integrating treatment efficacy and toxicity within a unified risk-stratification framework. In this setting, the key clinical question is not only whether a patient is likely to achieve pCR but also how the expected pathological benefit should be considered together with the risk of severe hematotoxicity. By integrating these two dimensions into a single utility-based stratification framework, the model provides a more decision-oriented representation of the expected efficacy–toxicity balance. Given the retrospective nature of the present study, this framework should not yet be regarded as a treatment-selection tool; rather, it may provide a basis for future prospective evaluation of risk-adapted treatment monitoring and supportive-care strategies.

Several limitations should be acknowledged. First, this was a retrospective study, and the external validation centers were located in the same city and belonged to the same university-affiliated healthcare system as the development center, which may limit the generalizability of the findings to other regions and independent healthcare systems. Second, despite restricting predictors to pretreatment information, heterogeneity remained in planned treatment exposure, including anthracycline and immunotherapy use, and treatment practices may have evolved during the study period. The use of complete-case data may also have introduced selection bias. Third, severe hematotoxicity was retrospectively ascertained from medical records, and PFS differences did not reach statistical significance in the external validation cohort, indicating that the prognostic value of the stratification requires further confirmation. Finally, the λ value was derived from data-driven optimization, and the linear utility function represents a simplified efficacy–toxicity trade-off. Individual patient preferences regarding the relative importance of pathological benefit and severe hematotoxicity were not formally elicited in this retrospective study and may substantially alter the clinically appropriate value of λ. Although sensitivity analyses using alternative λ values showed broadly consistent stratification patterns, the reference λ and corresponding cutoffs should therefore not be regarded as universal patient-level preferences. Future prospective studies should incorporate formal patient-preference assessment and validate the utility framework in broader and geographically independent populations.

## 5. Conclusions

This study developed and validated a TabNet-based multitask learning framework to jointly estimate pCR and severe hematotoxicity in patients with TNBC receiving platinum-based neoadjuvant therapy. The utility-based framework integrated efficacy and toxicity predictions into clinically interpretable benefit–hematotoxicity stratification. These findings support further prospective evaluation of the framework as a potential tool for individualized risk assessment and toxicity monitoring.

## Figures and Tables

**Table 1:** Patient characteristics.

	Training Cohort	Internal Validation Cohort	External Validation Cohort	
Variable	*n* = 1406	*n* = 351	*n* = 303	*p*
**Age (years)**	50.1 ± 10.6	51.0 ± 10.8	49.4 ± 10.8	0.179
**BMI (kg/m^2^)**	24.2 ± 3.5	24.4 ± 3.6	24.3 ± 3.4	0.564
**Hypertension, *n* (%)**				0.406
Yes	275 (19.6)	77 (21.9)	54 (17.8)	
No	1131 (80.4)	274 (78.1)	249 (82.2)	
**Diabetes, *n* (%)**				0.581
Yes	133 (9.5)	36 (10.3)	24 (7.9)	
No	1273 (90.5)	315 (89.7)	279 (92.1)	
**Clinical stage, *n* (%)**				0.730
Stage I	165 (11.7)	37 (10.5)	38 (12.5)	
Stage II	820 (58.3)	217 (61.8)	172 (56.8)	
Stage III	421 (29.9)	97 (27.6)	93 (30.7)	
**Anthracycline exposure, *n* (%)**				0.519
Yes	935 (66.5)	236 (67.2)	192 (63.4)	
No	471 (33.5)	115 (32.8)	111 (36.6)	
**Immunotherapy exposure, *n* (%)**				0.364
Yes	90 (6.4)	26 (7.4)	26 (8.6)	
No	1316 (93.6)	325 (92.6)	277 (91.4)	
**Tumor size, *n* (%)**				0.758
<20 mm	198 (14.1)	54 (15.4)	46 (15.2)	
20–50 mm	854 (60.7)	219 (62.4)	179 (59.1)	
>50 mm	354 (25.2)	78 (22.2)	78 (25.7)	
**Axillary lymph node, *n* (%)**				0.594
Negative	542 (38.5)	140 (39.9)	126 (41.6)	
Positive	864 (61.5)	211 (60.1)	177 (58.4)	
**Tumor margin, *n* (%)**				0.813
Well-defined	394 (28.0)	97 (27.6)	90 (29.7)	
Ill-defined	1012 (72.0)	254 (72.4)	213 (70.3)	
**Ki-67, *n* (%)**				0.636
<30%	530 (37.7)	135 (38.5)	120 (39.6)	
30–50%	608 (43.2)	150 (42.7)	117 (38.6)	
>50%	268 (19.1)	66 (18.8)	66 (21.8)	
**P53, *n* (%)**				0.195
Positive	945 (67.2)	218 (62.1)	200 (66.0)	
Negative	461 (32.8)	133 (37.9)	103 (34.0)	
**Vascularity, *n* (%)**				0.594
Low	512 (36.4)	120 (34.2)	115 (38.0)	
High	894 (63.6)	231 (65.8)	188 (62.0)	
**QTc interval, *n* (%)**				0.823
Normal	1056 (75.1)	261 (74.4)	232 (76.6)	
Borderline	233 (16.6)	65 (18.5)	48 (15.8)	
Prolonged	117 (8.3)	25 (7.1)	23 (7.6)	
**Heart rate, *n* (%)**				0.993
<60 bpm	92 (6.5)	24 (6.8)	18 (5.9)	
60–100 bpm	1204 (85.6)	300 (85.5)	262 (86.5)	
>100 bpm	110 (7.8)	27 (7.7)	23 (7.6)	

**Table 2:** Baseline laboratory characteristics of the study cohorts.

	Training Cohort	Internal Validation Cohort	External Validation Cohort	
Variable	*n* = 1406	*n* = 351	*n* = 303	*p*
ALT (U/L)	22.4 ± 18.4	23.7 ± 15.1	23.2 ± 17.4	0.451
AST (U/L)	22.9 ± 17.2	24.0 ± 17.4	22.5 ± 14.9	0.481
GGT (U/L)	24.7 ± 30.9	27.4 ± 31.3	25.5 ± 30.7	0.339
LDH (U/L)	185.0 ± 59.3	186.7 ± 59.7	186.1 ± 60.2	0.873
TBIL (μmol/L)	11.4 ± 3.1	11.7 ± 3.4	11.3 ± 3.0	0.157
DBIL (μmol/L)	3.4 ± 1.0	3.5 ± 1.1	3.5 ± 1.4	0.100
IDBIL (μmol/L)	8.5 ± 3.5	8.8 ± 3.0	8.5 ± 3.4	0.256
TP (g/L)	72.7 ± 4.6	72.4 ± 4.8	73.0 ± 4.5	0.215
ALB (g/L)	43.1 ± 2.9	43.0 ± 3.1	43.1 ± 2.9	0.665
GLB (g/L)	29.5 ± 3.1	29.9 ± 3.3	29.4 ± 3.0	0.059
A/G	1.48 ± 0.20	1.46 ± 0.21	1.48 ± 0.20	0.115
PALB (mg/L)	285.4 ± 58.4	287.8 ± 60.8	281.8 ± 57.4	0.423
BUN (mmol/L)	4.79 ± 2.00	4.86 ± 1.97	4.75 ± 1.95	0.756
CREA (μmol/L)	60.8 ± 13.9	61.6 ± 10.7	59.9 ± 12.4	0.290
UA (μmol/L)	285.5 ± 62.3	288.8 ± 67.2	281.7 ± 59.3	0.356
ALP (U/L)	76.5 ± 38.3	78.9 ± 39.7	75.2 ± 47.4	0.475
GLU (mmol/L)	5.26 ± 2.20	5.43 ± 1.98	5.22 ± 1.79	0.344
WBC (×10^9^/L)	6.20 ± 1.50	6.34 ± 1.60	6.09 ± 1.43	0.107
NEU (×10^9^/L)	3.90 ± 1.19	3.97 ± 1.26	3.82 ± 1.16	0.290
LYM (×10^9^/L)	1.80 ± 0.48	1.75 ± 0.46	1.82 ± 0.49	0.161
MON (×10^9^/L)	0.43 ± 0.12	0.44 ± 0.13	0.42 ± 0.11	0.154
EOS (×10^9^/L)	0.12 ± 0.06	0.12 ± 0.05	0.12 ± 0.06	0.795
BASO (×10^9^/L)	0.03 ± 0.01	0.03 ± 0.01	0.03 ± 0.01	0.977
Hb (g/L)	126.1 ± 13.1	125.0 ± 14.2	126.8 ± 12.8	0.205
RBC (×10^12^/L)	4.20 ± 0.34	4.17 ± 0.50	4.22 ± 0.33	0.273
Hct (%)	38.5 ± 3.0	38.2 ± 3.1	38.7 ± 2.9	0.107
PLT (×10^9^/L)	258.7 ± 58.0	259.9 ± 60.5	254.5 ± 55.7	0.438
Fibrinogen (g/L)	3.13 ± 0.57	3.20 ± 0.62	3.09 ± 0.56	0.051
D-dimer (mg/L FEU)	0.46 ± 1.29	0.47 ± 1.24	0.46 ± 1.27	0.977

**Table 3:** Predictive performance of MT-TabNet across study cohorts.

Outcome	AUC (95% CI)	Sensitivity	Specificity	PPV	NPV	Brier Score
**pCR**						
Training	0.874 (0.849–0.903)	0.787	0.796	0.634	0.893	0.145
Internal validation	0.842 (0.787–0.889)	0.762	0.782	0.612	0.879	0.158
External validation	0.819 (0.744–0.876)	0.746	0.747	0.57	0.867	0.169
**Severe hematotoxicity**						
Training	0.859 (0.845–0.888)	0.776	0.786	0.637	0.879	0.143
Internal validation	0.846 (0.797–0.896)	0.758	0.788	0.611	0.881	0.156
External validation	0.818 (0.757–0.871)	0.724	0.774	0.559	0.876	0.169

**Table 4:** Clinical outcomes by benefit group across study cohorts.

Cohort	*n* (%)	pCR, *n* (%)	*p*	Severe Hematotoxicity, *n* (%)	*p*	Favorable Outcome, *n* (%)	*p*
**Training**			<0.001		<0.001		<0.001
Low benefit	352 (25.0)	61 (17.3)		155 (44.0)		34 (9.7)	
Intermediate benefit	702 (49.9)	217 (30.9)		225 (32.1)		147 (20.9)	
High benefit	352 (25.0)	158 (44.9)		78 (22.2)		123 (34.9)	
**Internal validation**			<0.001		<0.001		<0.001
Low benefit	84 (23.9)	14 (16.7)		39 (46.4)		8 (9.5)	
Intermediate benefit	181 (51.6)	56 (30.9)		53 (29.3)		40 (22.1)	
High benefit	86 (24.5)	39 (45.3)		15 (17.4)		32 (37.2)	
**External validation**			<0.001		<0.001		<0.001
Low benefit	73 (24.1)	12 (16.4)		33 (45.2)		7 (9.6)	
Intermediate benefit	154 (50.8)	47 (30.5)		41 (26.6)		35 (22.7)	
High benefit	76 (25.1)	35 (46.1)		12 (15.8)		30 (39.5)	

## Data Availability

The datasets generated and/or analyzed in the current study are available from the corresponding author upon reasonable request.

## Supplementary Materials

The following supporting information can be downloaded at: https://www.mdpi.com/article/10.3390/cancers18172842/s1.
