# Supplementary material for: Benefit–Hematotoxicity Stratification in Patients with Triple-Negative Breast Cancer Receiving Platinum-Based Neoadjuvant Therapy: A Multitask Deep Learning Study
Source: Cancers (Basel). 2026 Sep 2;18(17):2842. doi: 10.3390/cancers18172842 (PMC13565154; doi:10.3390/cancers18172842)
Supplement: Supplementary file 1 [file cancers-18-02842-s001.zip › cancers-4442981-supplementary.pdf]

Supplementary Materials

Supplementary Table S1. Summary of missing data and reasons for missingness

**Missing data category**	**Specific missing item**	**Patients with missing data, n=28**	**Reason for missingness**
**Missing primary outcome data**			
pCR outcome	Postoperative pathological response assessment	7	Postoperative pathological response assessment was unavailable because of death before surgery (n = 1), failure to undergo surgery (n = 2), or treatment discontinuation (n = 4)
Severe hematotoxicity outcome	Severe hematotoxicity records	5	Treatment records or laboratory data were incomplete, preventing reliable ascertainment of the severe hematotoxicity outcome
**Missing baseline predictors**			
Pathological variables	Ki-67, P53	5	Pretreatment pathological reports were unavailable or the relevant variables were not recorded
Electrocardiographic variables	QTc interval, heart rate	4	Pretreatment electrocardiographic examination results or original reports were unavailable
Tumor and imaging variables	Tumor size, axillary lymph node status, tumor margin	4	Pretreatment imaging data or original imaging reports were incomplete or unavailable
Coagulation-related variables	D-dimer, fibrinogen	3	Pretreatment coagulation test results were unavailable

Note: Patients with missing outcome data or essential baseline predictors were excluded from the final analysis. Complete-case analysis was performed without imputation. pCR, pathological complete response; QTc, corrected QT interval.

Supplementary Table S2. Predictor timing and data sources for model development

**Predictor category**	**Variables**	**Source**	**Timing**

Demographic/comorbidity	Age, BMI, hypertension, diabetes	Clinical records	Pretreatment
Tumor characteristics	Stage, tumor size, axillary node status, tumor margin, vascularity	Clinical/imaging assessment	Pretreatment
Pathological markers	Ki-67, P53	Pretreatment pathology	Pretreatment
Laboratory variables	Liver/renal function, hematology, coagulation, etc.	Laboratory tests	Pretreatment
ECG variables	QTc, heart rate	Electrocardiography	Pretreatment
Planned treatment exposure	Planned anthracycline and immunotherapy	Pretreatment treatment plan	Pretreatment

Note: All candidate predictors were available before initiation of neoadjuvant therapy; no post-treatment response or hematotoxicity information was used for model development.

Supplementary Table S3. Hyperparameter search spaces for prediction models

Model	Prediction task	Hyperparameter	Search space	Final selected value
Ridge LR	pCR	C	0.001, 0.01, 0.1, 1, 10, 100	1
Ridge LR	Severe hematotoxicity	C	0.001, 0.01, 0.1, 1, 10, 100	0.1
RF	pCR	n_estimators	300, 500, 800, 1000	800
		max_depth	None, 4, 6, 8, 10, 12	8
		min_samples_split	2, 5, 10, 20	5
		min_samples_leaf	1, 2, 4, 8	2
		max_features	sqrt, log2, 0.5, 1.0	sqrt
RF	Severe hematotoxicity	n_estimators	300, 500, 800, 1000	800
		max_depth	None, 4, 6, 8, 10, 12	10
		min_samples_split	2, 5, 10, 20	5
		min_samples_leaf	1, 2, 4, 8	2
		max_features	sqrt, log2, 0.5, 1.0	sqrt
XGBoost	pCR	n_estimators	200, 400, 600, 800, 1000	600
		learning_rate	0.01, 0.03, 0.05, 0.10	0.05

		max_depth	2, 3, 4, 5, 6	3
		min_child_weight	1, 3, 5, 10	3
		subsample	0.6, 0.8, 1.0	0.8
		colsample_bytree	0.6, 0.8, 1.0	0.8
		gamma	0, 0.1, 0.3, 0.5	0.1
		reg_alpha	0, 0.01, 0.1, 1	0.01
		reg_lambda	0.1, 1, 5, 10	1
XGBoost	Severe hematotoxicity	n_estimators	200, 400, 600, 800, 1000	800
		learning_rate	0.01, 0.03, 0.05, 0.10	0.03
		max_depth	2, 3, 4, 5, 6	3
		min_child_weight	1, 3, 5, 10	3
		subsample	0.6, 0.8, 1.0	0.8
		colsample_bytree	0.6, 0.8, 1.0	0.8
		gamma	0, 0.1, 0.3, 0.5	0.1
		reg_alpha	0, 0.01, 0.1, 1	0.01
		reg_lambda	0.1, 1, 5, 10	1
ST-TabNet	pCR	n_d = n_a	8, 16, 32, 64	32
		n_steps	3, 4, 5, 6, 7	5
		gamma	1.0, 1.2, 1.3, 1.5, 1.8, 2.0	1.3
		lambda_sparse	0, 1e−5, 1e−4, 1e−3, 1e−2	1e−4
		learning rate	0.005, 0.01, 0.02, 0.03, 0.05	0.02
		batch size	128, 256, 512	256
		virtual batch size	32, 64, 128	64
ST-TabNet	Severe hematotoxicity	n_d = n_a	8, 16, 32, 64	32
		n_steps	3, 4, 5, 6, 7	4

		gamma	1.0, 1.2, 1.3, 1.5, 1.8, 2.0	1.3
		lambda_sparse	0, 1e−5, 1e−4, 1e−3, 1e−2	1e−4
		learning rate	0.005, 0.01, 0.02, 0.03, 0.05	0.02
		batch size	128, 256, 512	256
		virtual batch size	32, 64, 128	64
MT-TabNet	Joint pCR and severe hematotoxicity	n_d = n_a	8, 16, 32, 64	32
		n_steps	3, 4, 5, 6, 7	5
		gamma	1.0, 1.2, 1.3, 1.5, 1.8, 2.0	1.3
		lambda_sparse	0, 1e−5, 1e−4, 1e−3, 1e−2	1e−4
		learning rate	0.005, 0.01, 0.02, 0.03, 0.05	0.02
		batch size	128, 256, 512	256
		virtual batch size	32, 64, 128	64

Supplementary Table S4. Pairwise standardized mean differences of baseline clinical characteristics

**Variable**	**Training vs Internal validation**	**Training vs External validation**	**Internal vs External validation**
Age (years)	0.080	0.063	0.142
BMI (kg/m²)	0.061	0.031	0.031
Hypertension	0.059	0.045	0.103
Diabetes	0.027	0.055	0.081
Clinical stage	0.072	0.034	0.105
Anthracycline exposure	0.016	0.066	0.081
Immunotherapy exposure	0.040	0.083	0.043
Tumor size	0.073	0.038	0.084
Axillary lymph node	0.027	0.062	0.035
Tumor margin	0.009	0.037	0.046

Ki-67	0.016	0.099	0.094
P53	0.107	0.026	0.081
Vascularity	0.047	0.032	0.078
QTc interval	0.064	0.036	0.072
Heart rate	0.012	0.027	0.037

Supplementary Table S5. Pairwise standardized mean differences of baseline laboratory characteristics

Variable	Training vs Internal validation	Training vs External validation	Internal vs External validation
ALT	0.075	0.04	0.033
AST	0.062	0.024	0.09
GGT	0.087	0.028	0.059
LDH	0.028	0.019	0.009
TBIL	0.099	0.035	0.134
DBIL	0.074	0.112	0.048
IDBIL	0.102	0.004	0.099
TP	0.074	0.062	0.135
ALB	0.052	0.021	0.032
GLB	0.133	0.015	0.150
A/G	0.13	0.060	0.066
PALB	0.042	0.061	0.102
BUN	0.036	0.021	0.057
CREA	0.058	0.069	0.141
UA	0.05	0.063	0.112
ALP	0.061	0.029	0.084
GLU	0.081	0.02	0.111

WBC	0.089	0.074	0.162
NEU	0.053	0.07	0.122
LYM	0.101	0.038	0.138
MON	0.061	0.092	0.15
EOS	0.005	0.043	0.042
BASO	0.013	0.006	0.006
Hb	0.079	0.056	0.134
RBC	0.064	0.055	0.108
Hct	0.095	0.066	0.162
PLT	0.02	0.075	0.093
Fibrinogen	0.111	0.073	0.181
D-dimer	0.013	0.001	0.012

Supplementary Table S6. Detailed predictive performance and calibration metrics of the MT-TabNet model across the study cohorts

Outcome	Sensitivity (95% CI)	Specificity (95% CI)	PPV (95% CI)	NPV (95% CI)	Brier score (95% CI)	Calibration intercept (95% CI)	Calibration slope (95% CI)
**pCR**							
Training	0.787 (0.746–0.823)	0.796 (0.769–0.820)	0.634 (0.592–0.673)	0.893 (0.871–0.912)	0.145 (0.133–0.157)	0.01 (−0.07–0.09)	0.98 (0.86–1.10)
Internal validation	0.762 (0.674–0.833)	0.782 (0.726–0.829)	0.612 (0.527–0.689)	0.879 (0.829–0.916)	0.158 (0.133–0.183)	−0.04 (−0.21–0.13)	0.94 (0.71–1.18)
External validation	0.746 (0.649–0.823)	0.747 (0.684–0.802)	0.570 (0.481–0.654)	0.867 (0.808–0.908)	0.169 (0.141–0.197)	−0.07 (−0.26–0.12)	0.90 (0.66–1.15)
**Severe hematotoxicity**							
Training	0.776 (0.735–0.812)	0.786 (0.758–0.811)	0.637 (0.596–0.675)	0.879 (0.855–0.900)	0.143 (0.131–0.155)	0.02 (−0.06–0.10)	0.99 (0.87–1.11)
Internal validation	0.758 (0.669–0.829)	0.788 (0.733–0.835)	0.611 (0.525–0.691)	0.881 (0.832–0.918)	0.156 (0.131–0.181)	0.01 (−0.16–0.18)	0.96 (0.73–1.21)
External validation	0.724 (0.621–0.807)	0.774 (0.714–0.825)	0.559 (0.466–0.650)	0.876 (0.822–0.915)	0.169 (0.141–0.197)	0.08 (−0.12–0.28)	0.89 (0.64–1.15)

Supplementary Table S7. Comparison of model discrimination for pCR prediction across study cohorts

Cohort	Model	AUC (95% CI)	DeLong P vs MT-TabNet
Training	LR	0.752 (0.721–0.781)	<0.001
	RF	0.823 (0.796–0.848)	<0.001
	XGBoost	0.826 (0.799–0.851)	<0.001
	ST-TabNet	0.876 (0.853–0.897)	0.781
	MT-TabNet	0.874 (0.849–0.903)	Reference
Internal validation	LR	0.724 (0.660–0.783)	0.003
	RF	0.787 (0.729–0.840)	0.126
	XGBoost	0.822 (0.768–0.872)	0.472
	ST-TabNet	0.837 (0.784–0.885)	0.824
	MT-TabNet	0.842 (0.787–0.889)	Reference
External validation	LR	0.657 (0.585–0.724)	<0.001
	RF	0.772 (0.708–0.830)	0.109
	XGBoost	0.810 (0.750–0.865)	0.759
	ST-TabNet	0.823 (0.764–0.877)	0.846
	MT-TabNet	0.819 (0.744–0.876)	Reference

Supplementary Table S8. Comparison of model discrimination for severe hematotoxicity prediction across study cohorts

Cohort	Model	AUC (95% CI)	DeLong P vs MT-TabNet
Training	LR	0.693 (0.661–0.724)	<0.001
	RF	0.775 (0.746–0.802)	<0.001
	XGBoost	0.811 (0.784–0.836)	<0.001
	ST-TabNet	0.859 (0.835–0.881)	0.994
	MT-TabNet	0.859 (0.845–0.888)	Reference

Internal validation	LR	0.678 (0.613–0.739)	<0.001
	RF	0.745 (0.683–0.802)	0.009
	XGBoost	0.778 (0.720–0.832)	0.041
	ST-TabNet	0.829 (0.776–0.877)	0.392
	MT-TabNet	0.846 (0.797–0.896)	Reference
External validation	LR	0.683 (0.610–0.751)	0.005
	RF	0.757 (0.689–0.819)	0.146
	XGBoost	0.772 (0.706–0.832)	0.233
	ST-TabNet	0.825 (0.765–0.879)	0.763
	MT-TabNet	0.818 (0.757–0.871)	Reference

Supplementary Table S9. Quantification of task-level transfer between MT-TabNet and ST-TabNet models

Outcome	ST-TabNet AUC	MT-TabNet AUC	ΔAUC (95% CI)	ST-TabNet Brier score	MT-TabNet Brier score	ΔBrier (95% CI)
**pCR**						
Repeated cross-validation	0.849 ± 0.010	0.852 ± 0.011	+0.003 (−0.004 to 0.010)	0.153 ± 0.006	0.151 ± 0.006	+0.002 (−0.002 to 0.006)
Internal validation	0.837	0.842	+0.005 (−0.012 to 0.022)	0.161	0.158	+0.003 (−0.003 to 0.009)
External validation	0.823	0.819	−0.004 (−0.024 to 0.016)	0.168	0.169	−0.001 (−0.008 to 0.006)
**Severe hematotoxicity**						
Repeated cross-validation	0.835 ± 0.012	0.838 ± 0.013	+0.003 (−0.005 to 0.011)	0.160 ± 0.007	0.158 ± 0.007	+0.002 (−0.002 to 0.006)
Internal validation	0.829	0.846	+0.017 (0.001 to 0.033)	0.162	0.156	+0.006 (0.000 to 0.012)
External validation	0.825	0.818	−0.007 (−0.028 to 0.014)	0.167	0.169	−0.002 (−0.009 to 0.005)

Note: ΔAUC was calculated as MT-TabNet minus ST-TabNet, whereas ΔBrier was calculated as ST-TabNet minus MT-TabNet; therefore, positive values indicate better performance of MT-TabNet for both metrics.

Supplementary Table S10. Sensitivity analysis of utility-based stratification across alternative λ values

λ	Lower cutoff	Upper cutoff	Low / Intermediate / High, n	ΔpCR, High−Low	Δ severe hematotoxicity, Low−High	Δ favorable outcome, High−Low
0.5	0.07	0.4	68 / 158 / 77	29.30%	21.50%	30.00%
1	−0.02	0.31	71 / 156 / 76	30.60%	23.70%	30.90%
1.255*	−0.08	0.26	73 / 154 / 76	29.70%	29.40%	29.90%
1.5	−0.13	0.21	76 / 151 / 76	26.30%	34.20%	30.30%
2	−0.23	0.12	82 / 147 / 74	18.50%	36.50%	27.00%

Note: Cutoffs for each λ were derived from the training cohort and applied unchanged to the external validation cohort. Δ values indicate High−Low differences for pCR and favorable outcome and Low−High differences for severe hematotoxicity. Favorable outcome was defined as pCR without severe hematotoxicity. *λ = 1.255 was the reference value.
